# Synthesis, Screening and Characterization of Novel Potent Arp2/3 Inhibitory Compounds Analogous to CK-666

**DOI:** 10.3389/fphar.2022.896994

**Published:** 2022-05-30

**Authors:** Artem I. Fokin, Roman N. Chuprov-Netochin, Alexander S. Malyshev, Stéphane Romero, Marina N. Semenova, Leonid D. Konyushkin, Sergey V. Leonov, Victor V. Semenov, Alexis M. Gautreau

**Affiliations:** ^1^ CNRS UMR7654, Ecole Polytechnique, Institut Polytechnique de Paris, Palaiseau, France; ^2^ Department of Molecular and Bio Physics, Moscow Institute of Physics and Technology, Dolgoprudny, Russia; ^3^ Lomonosov Moscow State University, Faculty of Medicine, Moscow, Russia; ^4^ Dukhov Research Institute of Automatics (VNIIA), Moscow, Russia; ^5^ N. K. Koltzov Institute of Developmental Biology RAS, Moscow, Russia; ^6^ N. D. Zelinsky Institute of Organic Chemistry RAS, Moscow, Russia; ^7^ Center of Molecular and Cellular Biology, Skolkovo Institute of Science and Technology, Moscow, Russia

**Keywords:** Arp2/3, branched actin, Arp2/3 inhibitors, CK-666, actin polymerization

## Abstract

Branched actin networks polymerized by the Actin-related protein 2 and 3 (Arp2/3) complex play key roles in force generation and membrane remodeling. These networks are particularly important for cell migration, where they drive membrane protrusions of lamellipodia. Several Arp2/3 inhibitory compounds have been identified. Among them, the most widely used is CK-666 (2-Fluoro-N-[2-(2-methyl-1H-indol-3-yl)ethyl]-benzamide), whose mode of action is to prevent Arp2/3 from reaching its active conformation. Here 74 compounds structurally related to CK-666 were screened using a variety of assays. The primary screen involved EdU (5-ethynyl-2′-deoxyuridine) incorporation in untransformed MCF10A cells. The resulting nine positive hits were all blocking lamellipodial protrusions and cell migration in B16-F1 melanoma cells in secondary screens, showing that cell cycle progression can be a useful read-out of Arp2/3 activity. Selected compounds were also characterized on sea urchin embryos, where Arp2/3 inhibition yields specific phenotypes such as the lack of triradiate spicules and inhibition of archenteron elongation. Several compounds were filtered out due to their toxicity in cell cultures or on sea urchin development. Two CK-666 analogs, **59** (N-{2-[5-(Benzyloxy)-2-methyl-1H-indol-3-yl] ethyl}-3-bromobenzamide) and **69** (2,4-Dichloro-N-[2-(7-chloro-2-methyl-1H-indol-3-yl) ethyl]-5-[(dimethylamino) sulfonyl] benzamide), were active in all assays and significantly more efficient *in vivo* than CK-666. These best hits with increased *in vivo* potency were, however, slightly less efficient *in vitro* than CK-666 in the classical pyrene-actin assay. Induced-fit docking of selected compounds and their possible metabolites revealed interaction with Arp2/3 that suppresses Arp2/3 activation. The data obtained in our screening validated the applicability of original assays for Arp2/3 activity. Several previously unexplored CK-666 structural analogs were found to suppress Arp2/3 activation, and two of them were identified as Arp2/3 inhibitors with improved *in vivo* efficiency.

## Introduction

Arp2/3 is a ubiquitous heptameric complex, which generates branched actin structures that remodel cell membranes through force generation (Pollard, 2007). Arp2/3 activation is triggered by a conformational change, when Nucleation Promoting Factors (NPFs) bind to two sites on the Arp2/3 complex (Zimmet et al., 2020; Gautreau et al., 2021). Four families of NPFs have been discovered in mammalian genomes. They activate Arp2/3 at different subcellular locations (Molinie and Gautreau, 2018). For example, the two NPFs WAVE (Wiskott-Aldrich syndrome and verprolin-homologous protein) and WASH (Wiskott-Aldrich syndrome protein and SCAR homologue) activate Arp2/3 at the cell cortex or at endosomal subdomains to generate lamellipodial protrusions and endosomal sorting of receptors, respectively. Arp2/3 activation involves bringing into proximity the two Actin-Related Proteins Arp2 and Arp3, in a “short-pitch” conformation that mimics the barbed end of an actin filament (Goley et al., 2004; Rodal et al., 2005; Rouiller et al., 2008; Smith et al., 2013).

Several classes of compounds that inhibit Arp2/3 have been discovered. Two 2-methyl-triptamine derivatives, 2-fluoro-*N*-[2-(2-methyl-1*H*-indol-3-yl)ethyl] benzamide (CK-666) and to a lesser extent its analog CK-636, where 2-fluorobenzamide fragment is substituted by 2-thiophenecarboxamide, are the most widely used. They bind to the Arp2/3 complex at the interface between its Arp2 and Arp3 subunits and prevent the Arp2/3 to reach the short pitch conformation (Nolen et al., 2009; Baggett et al., 2012; Hetrick et al., 2013). Two other compounds, CK-548/869 (Nolen et al., 2009), bind to a hydrophobic pocket within the Arp3 subunit, and also destabilize the short-pitch conformation of the complex (Hetrick et al., 2013). Another compound occupying the same Arp3 hydrophobic pocket, oleanane triterpenoid–cyano-3,12-dioxooleana-1,9-dien-28-oicacid (CDDO), was also found to inhibit Arp2/3 activation (To et al., 2010). Finally, two other Arp2/3 inhibitory compounds were found by repositioning FDA-approved drugs. The antitussive benproperine (Jin et al., 2019) and the antipsychotic pimozide (Choi et al., 2019) both inhibit Arp2/3 by binding to the ARPC2 subunit of the Arp2/3 complex.

Baggett and others improved the Arp2/3 binding ability of CK-666 through *in silico* docking (Baggett et al., 2012). A compound containing an additional hydroxyl at the fourth position of the benzene ring was more active *in vitro*, but has not yet been validated *in vivo*. The impact of different substitutions in the fluorobenzene and the indole rings of CK-666 was not studied yet. Since CK-666 inhibits Arp2/3 activation with IC_50_ of about 50 μM, increasing the efficiency of Arp2/3 inhibitors can be considered as important for their further therapeutic use. The Arp2/3 complex and complexes containing the NPFs WAVE and WASH are overexpressed in a variety of cancers (Molinie and Gautreau, 2018). Arp2/3 overexpression is associated with high-grade tumors and poor prognosis in colorectal, liver, lung and breast cancers. Tumor cells overexpressing the Arp2/3 complex subunits or activating the Arp2/3 complex through the *RAC1* oncogene are sensitive to Arp2/3 inhibition (Liu et al., 2013; Molinie et al., 2019). These data suggest that Arp2/3 inhibitory compounds might be useful in isolation or in combination for precision medicine of cancer. Here we screened compounds exploring the CK-666 scaffold and characterized CK-666 analogs using *in vitro* and *in vivo* assays. We reveal a couple of compounds with increased *in vivo* efficiency.

## Materials and Methods

### Chemistry

**Scheme 1:**
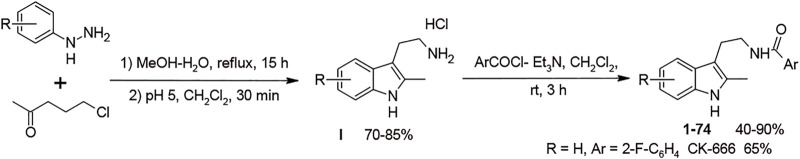
Synthesis of substituted 2-methyltryptamines **1–74** and CK-666.

Melting points were measured on a Boetius melting point apparatus and were uncorrected. 1H NMR spectra were recorded on a Bruker DRX-500 instrument [working frequencies of 500.13 MHz (1H) and 125.76 MHz (13C)]. Chemical shifts were stated in parts per million (ppm) and referenced to the appropriate NMR solvent peaks. Spin-spin coupling constants (J) were reported in Hertz (Hz). Low resolution mass spectra (m/z) were recorded on a Finnigan MAT/INCOS 50 mass spectrometer at 70 eV using direct probe injection. Elemental analysis was performed on the automated Perkin-Elmer 2400 CHN microanalyzer. Flash chromatography was carried out on silica gel (Acros, 0.035–0.070 mm, 60 Å). TLC was performed on Merck 60 F254 plates. Solvents, 2-methyltriptamine, and acid chloroanhydrides were purchased from Chemical Block Ltd. (www.chemical-block.com) at the highest commercial quality and used as received. Compounds **1**–**74** (Supplementary Table S1) were purchased from Chemical Block Ltd. (www.chemical-block.com), where they were synthesized according to Scheme 1. Intermediate 2-methyltryptamines **I** were synthesized from arylhydrazine and 5-chloropenthanone-2 by Fisher-Grandberg reaction (Scheme 1). Further acylation of 2-methyltryptamines **I** using the respective chloroanhydrides of benzoic and heterocyclic acids afforded the target molecules **1**–**74** and specific inhibitor of Arp2/3 complex CK-666. Their purity and structure were further confirmed by ^1^H NMR and mass spectrometry and elemental analysis (Supplementary Data Sheet S1).

### General Procedure for the Preparation of CK-666

A solution of Et_3_N (3.06 g, 30.3 mmol) and 2-methyltryptamine (1.97 g, 10.1 mmol) in dry CH_2_Cl_2_ (30 ml) was stirred for 1 h at 20°C, cooled until 0°C, and then chloro-anhydride (11.5 mmol) in dry CH2Cl2 (10 ml) was added dropwise. The reaction mixture was stirred for 3 h at room temperature, washed with water, dried and evaporated. The product was separated by column chromatography (EtOAc/petroleum ether = 1/3, R_*f*_ = 0.3) and dried in vacuum (2 mm Hg) at 35–40°С to afford the target pure material.

### Cell Cultures

The immortalized epithelial cell line from human breast, MCF10A was obtained from the Institut Curie collection of breast cell lines, organized and maintained by Thierry Dubois in Paris. MCF10A cells were cultured in DMEM/F12 medium (Gibco) supplemented with 5% horse serum (Sigma), 100 ng/ml cholera toxin (Sigma), 20 ng/ml epidermal growth factor (Sigma), 0.01 mg/ml insulin (Sigma), 500 ng/ml hydrocortisone (Sigma) and 100 U/ml penicillin/streptomycin (Gibco). B16-F1 mouse melanoma cells were obtained from Klemens Rottner (Technische Universität Braunschweig, Germany). B16-F1 cells were grown in DMEM supplemented with 10% fetal calf serum (Invitrogen) and 100 U/ml penicillin/streptomycin (Gibco). Cells were incubated in a humidified atmosphere with 5% CO_2_.

### Cell Viability and Cell Cycle Progression

Cell viability was assessed using the CellTiter 96^®^ AQueous One Solution Cell Proliferation kit (Promega) on a Multiscan Spectrum spectrophotometer (Thermo LifeSciences), with absorbance read at 490 nm.

For cell cycle progression using EdU, MCF10A cells were seeded in 384-well plates at a density of 800 cells per well. After spreading, cells were treated for 18 h with compounds in DMSO (final DMSO concentration did not exceed 0.5%). EdU from kit C10337 (ThermoFischer Scientific, Waltham, MA, United States) was then added to the cells at 10 μM and incubated for 2.5 h. Cells were fixed in 4% paraformaldehyde for 30 min at room temperature. Click-iT reaction was performed for 1 h at room temperature, protected from light, with 5 µM Alexa Fluor 488 azide in (100 mM Tris, 1 mM CuSO4, 50 mM Ascorbic Acid, pH 8.5). Nuclei were counterstained with Hoechst 33,342 (Thermo Fisher Scientific). Imaging and analysis were performed using the ImageXpress Micro XL High-Content Screening System (Molecular Devices LLC, San Jose, CA, United States). IC50 values were determined by sigmoidal curve fitting using GraphPad Prism six software.

### Lamellipodium Formation and Cell Migration

Lyophilized compounds were resuspended with fresh DMSO from sealed vials (cell biology grade). Compounds, stored at +4°C, were used within a week. B16-F1 cells were seeded onto glass coverslips coated for 1 h with 25 μg/ml laminin (Sigma) in (50 mM Tris, 150 mM NaCl, pH 7.4). Tested compounds, CK-666 and its inactive control, CK-689 (N-(2-(1H-Indol-3-yl)ethyl)-2-methoxy-acetamide) (Merck) were added to cells for 1 h, then lamellipodia were promoted for 20 min in complete medium with AlCl_3_ and NaF at the final concentration of 50 μM and 30 mM, respectively. Cell were then processed for immunofluorescence.

For the wound healing, B16-F1 cells were seeded onto 8-well Ibidi dishes coated with laminin, and grown to confluence. Wounds in the cell monolayer were obtained by scratching the monolayer with a pipet tip. Detached cells were immediately washed away and new medium containing the various compounds was added. Cells were then used for videomicroscopy.

### Immunofluorescence

Cells were fixed in 3.2% PFA on PBS, then quenched with 50 mM NH_4_Cl and permeabilised with 0.5% Triton X-100. After blocking in 2% BSA cells were incubated with antibodies (1–5 μg/ml for the primary, 5 μg/ml for the secondary). Total actin in cells was visualized by phalloidin staining. Nuclei were stained with 0.3 μg/ml DAPI (Roche). Coverslips were mounted in Dako Fluorescence Mounting Medium (Agilent). For immunofluorescence, ARPC2 rabbit polyclonal antibody (Millipore, 07–227), Cortactin mouse monoclonal 4F11 (Millipore, 05–180) were used. Secondary goat anti-mouse and anti-rabbit antibodies conjugated with Alexa Fluor 555 and 647 respectively were from Life Technologies. Phalloidin conjugated with Alexa Fluor 488 was from Tebu-bio.

### Microscopy and Image Analysis

Videomicroscopy was performed on an inverted Axio Observer microscope (Zeiss) equipped with a Pecon Zeiss incubator XL multi S1 RED LS (Heating Unit XL S, Temp module, CO_2_ module, Heating Insert PS and CO_2_ cover), Definite focus module and a Hamamatsu camera C10600 Orca-R2. Phase contrast images were taken every 30 min for 24 h using the 10X/0.25 Plan-Apochromat air objective. For immunofluorescence, slides were observed and imaged with the 63X/1.4 Plan-Apochromat oil objective. Fluorophores were excited with the HBO 100 W lamp (Osram) installed in the HXP 120 Metal halide fluorescence light source (Zeiss).

Image analysis was performed in ImageJ or FIJI software. To measure wound healing, boundaries of the wound were drawn manually at different time points, then wound size was measured. A cell was counted as displaying a lamellipodium if a cortactin positive concave protrusion wider than the major diameter of the nucleus was displayed.

### Pyrene-Actin Polymerization Assay

Actin was purified from rabbit muscle and was pyrenyl-labeled (Carlier et al., 1984; Malm, 1984). Arp2/3 was from Cytoskeleton, Inc. VCA domain of N-WASP was purified as previously described (Clainche et al., 2003). Pyrene-actin polymerization assays were performed as previously (Romero et al., 2007). Briefly, CaATP-actin was converted into MgATP-actin in (5 mM Tris, 0.2 mM ATP, 0.1 mM CaCl_2_, 1 mM DTT, 1 mM MgCl_2_, and 0.2 mM EGTA, pH 7.6) and kept on ice for no more than 1 h. Actin polymerization was monitored in (5 mM Tris, 50 mM KCl, 0.2 mM ATP, 0.1 mM CaCl_2_, 1 mM DTT, 1 mM MgCl_2_, 0.2 mM EGTA, pH 7.6) in the presence of 2% DMSO alone or 2% DMSO containing the appropriated compound concentration. The increase in fluorescence of 10% pyrenyl-labeled actin was monitored using a Safas spectrofluorimeter (λ_exc_ = 366 nm, λ_em_ = 407 nm). The maximum rate of actin polymerization was derived from the maximum slope of the polymerization curve multiplied by [total polymer]/(RFUmax—RFUmin) [total polymer] is the total actin minus the critical concentration (0.1 μM) and RFU, Relative Fluorescence Unit. The maximum elongation rates of actin polymerization were plotted as a function of the concentration of the different compounds. For CK-666, **59**, **69** and **71**, curves were fitted with the following equation to determine the IC50.

$$M_{rate}=\;\;R_{max}-\frac{R_{max}-R_{min}}{1+\frac{IC_{50}}{\left(Compound\;Conc.\right)}}$$

where M_rate_ is the measured maximum rate of actin polymerization at a given compound concentration, R_max_ is the maximum rate of actin polymerization with no compound, and R_min_ is the maximum rate of actin polymerization at saturating concentration of compound.

The curve of compound **15** was fitted with a linear decay. The IC50 was determined at half inhibition of actin polymerization.

### Sea Urchin Embryos

The procedure was described in detail in Semenova et al., 2018. Briefly, adult sea urchins, *Paracentrotus lividus L* (Echinidae), were collected from the Mediterranean Sea on the Cyprus coast and kept in an aerated seawater tank. Gametes were obtained by intracoelomic injection of 0.5 M KCl. Eggs were washed with filtered seawater and fertilized by adding drops of diluted sperm. Embryos were cultured at room temperature under gentle agitation with a motor-driven plastic paddle (60 rpm) in filtered seawater. Embryos were observed with a Biolam light microscope (LOMO, St. Petersburg, Russian Federation). For treatment with test compounds, 5 ml aliquots of embryo suspension were transferred to six-well plates and incubated at a concentration of 1,000–2,000 embryos/ml. For images, sedimented embryos were concentrated in the center of the well by gentle rotation of the culture plate, and 200 μl aliquots containing several hundred embryos were used to take pictures (up to 20 images from different fields). Embryos selected for Figure 4 were representative of the population.

Stock solutions of compounds were prepared in DMSO at 50–250 mM concentration followed by a 10-fold dilution with 96% EtOH. This procedure enhanced the solubility of the test compounds in the salty seawater. Maximal tolerated concentrations of DMSO and EtOH in the assay were determined to be 0.05 and 1%, respectively. Higher concentrations of either DMSO (>0.1%) or EtOH (>1%) caused nonspecific alterations and retardation of the sea urchin embryo development independent of the treatment stage. Maximum solubility of compounds **15**, **59**, **69**, and **71** was about 5–20 mM in seawater, since visible crystals formed at higher concentrations.

The effects were assessed after exposure of fertilized eggs (8–15 min post fertilization, 45–55 min before the first mitotic cycle completion) to 2-fold decreasing concentrations of the compound. Embryonic development was monitored until the beginning of active feeding at four-arm pluteus stage (33–36 h postfertilization). Developmental abnormalities were estimated quantitatively as minimal effective concentration (MEC), resulting in inhibition of archenteron elongation or skeletal spicule formation.

Microphotographs were obtained using an AmScope binocular microscope with an MU500 digital camera (United Scopes LLC, Irvine, CA, United States). The embryos were immobilized with 5-[(6,7-dimethoxy-1,3-benzodioxol-5-yl)methyl]-3-(4-methoxyphenyl)-4,5-hydroisoxazole] at a concentration of 2 μM for 20 min (Semenova et al., 2008).

Experiments with sea urchin embryos fulfill the requirements of biological ethics: artificial spawning does not cause animal death, embryos develop outside the female organism, and both post spawned adult sea urchins and the excess of intact embryos were returned to the sea, their natural habitat.

### Molecular Modeling Using Induced-fit Docking

The 2.48 Å resolution of crystal structure of bovine Arp2/3 complexed with CK-666 (Nolen et al., 2009) was retrieved from the PDB code 3UKR and optimized using the Protein Preparation Wizard in Maestro (Schrödinger, 2021) adding bond orders and hydrogen atoms to the crystal structure using the OPLS3e force field (Roos et al., 2019). Prime was used to fix missing residues or atoms in the protein. PROPKA was used to check for the protonation state of ionizable protein groups at pH 7.0. Hydrogen bonds were optimized through the reorientation of hydroxyl bonds, thiol groups, and amide groups. In the end, the system was minimized with a value of convergence of the RMSD of 0.3 Å. Ligands were prepared using LigPrep with the OPLS3e force field.

Induced-fit docking (IFD) models conformational changes induced by ligand binding (Sherman et al., 2006). The steps used are detailed in (Tutone et al., 2019; Allegra et al., 2021). The grid box for the binding site was built considering the co-crystallized ligand CK-666 as a centroid, while other parameters were kept as default. The most favorable binding poses of ligands in Arp2/3 complex were selected based on the IFD score and visual inspection of binding modes. Selected complexes were visualized using the Ligand Interactions module in Maestro to check interactions between ligands and residues of the ligand binding pocket.

## Results

### Potential Arp2/3 Inhibitory Compounds Revealed Through a Primary Screen on Cell Cycle Progression

**FIGURE 1:**
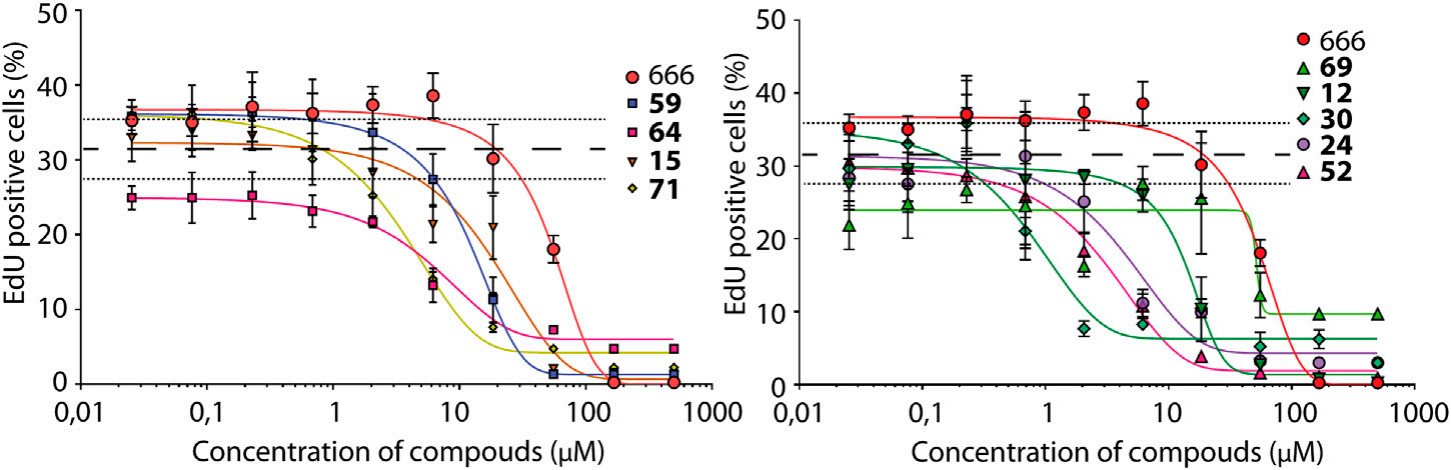
Screening of Arp2/3 inhibitors in MCF10A cells based on EdU incorporation. CK-666 and nine out of 74 tested analogs block cell cycle progression in a dose-dependent manner. Mean ± S.D. of three technical repeats. Two biological replicates with three technical repeats were performed and gave similar results. Only one is displayed. Dashed and dotted lines represent Mean ± SD of control cells treated with DMSO.

In the chemical library of Chemical Block Ltd. 74 compounds featuring the same 2-methyl triptamine skeleton as the canonical Arp2/3 inhibitor CK-666 were identified. To efficiently screen these compounds, we took advantage of the recent observation that Arp2/3 activity is required for cell cycle progression in untransformed cells (Molinie et al., 2019). The Arp2/3 inhibitor CK-666 blocks MCF10A cells in G1 phase. This defect translates into a lack of incorporation of EdU, a thymidine analog, into DNA during its replication in S phase of mitotic cycle. EdU nuclear labeling can be easily scored using automated microscopy by click chemistry with Hoechst counterstaining of all nuclei (Supplementary Figure S1). We tested in parallel all compounds with concentration values ranging from 0.025 to 100 μM. Nine molecules **12**, **15**, **24**, **30**, **52**, **59**, **64**, **69** and **71** were scored positive in this assay. They inhibited EdU incorporation more efficiently than CK-666 in a dose-dependent manner (Figure 1). Their chemical structures and rough IC_50_ values are summarized in Table 1. The other compounds showed little or no effect.

**TABLE 1:** Nine compounds that were more active than CK-666 in the EdU incorporation assay. IC_50_ is the concentration required for 50% decrease of EdU-positive cells calculated from the graphs displayed in Figure 1B.

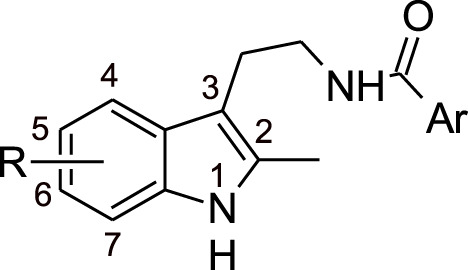
Compound ID	IC_50_ (μM)	R	Ar
30	0.6956	5-Me	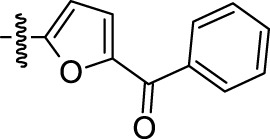
52	3.169	5-F_3_CO	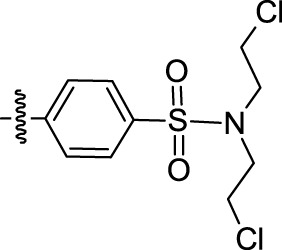
71	3.575	7-F_3_C	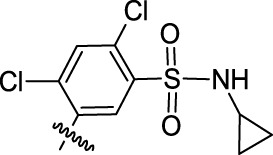
24	4.015	5-Me	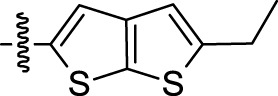
64	6.661	5-F	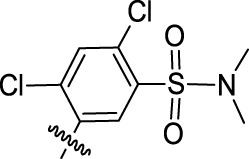
59	11.48	5-BnO	3-Br-C_6_H_4_
12	13.75	H	2-Cl-5-NO_2_-C_6_H_3_
15	19.98	H	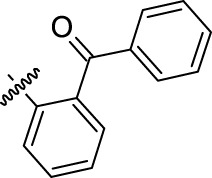
69	50.50	7-Cl	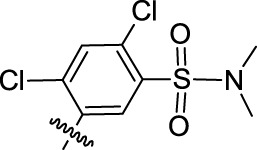
CK-666	56.29	H	2-F-C_6_H_4_

### Effect of CK-666 Analogs on Lamellipodium Formation and Cell Migration

**FIGURE 2:**
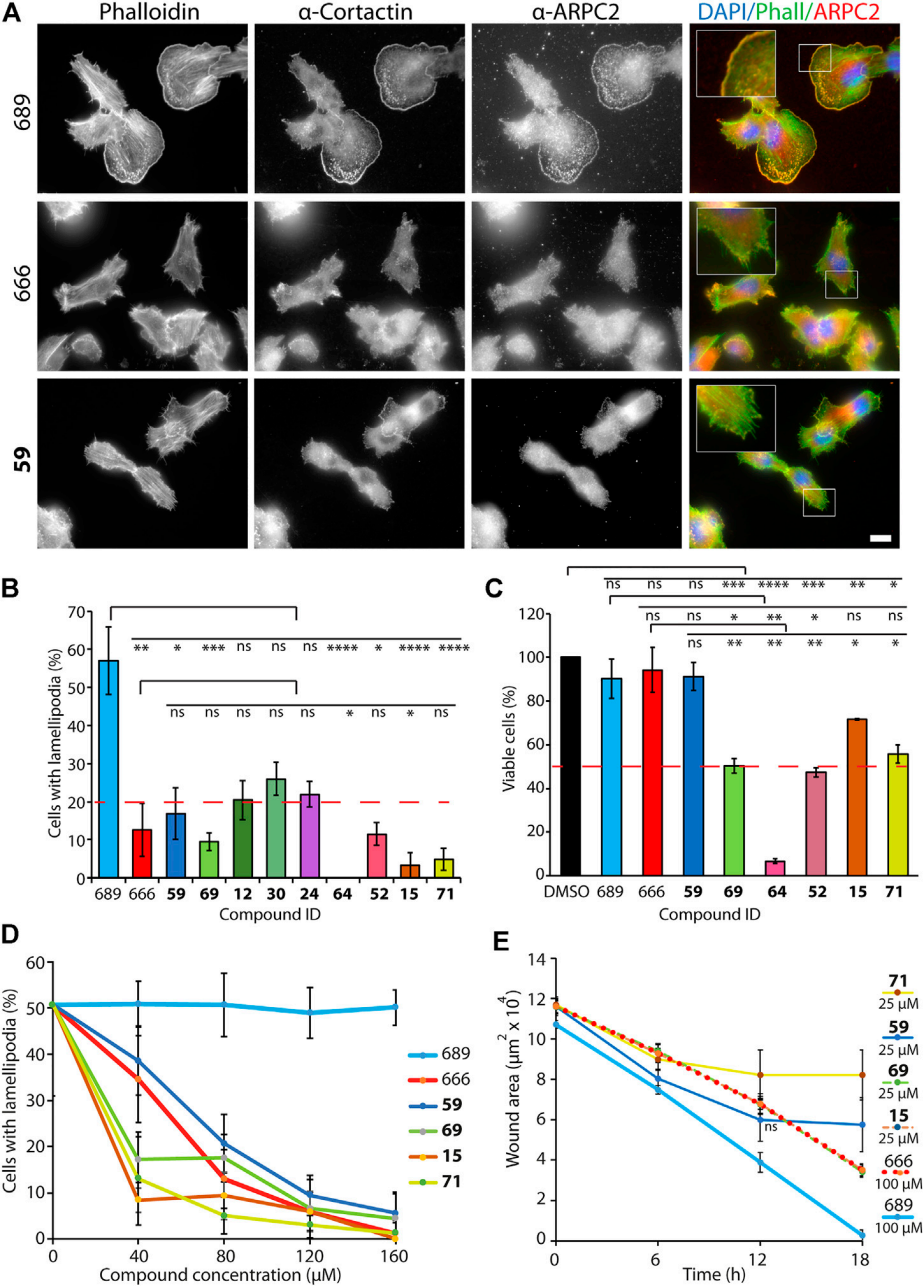
Effect of selected CK-666 analogs on lamellipodium protrusion and cell migration in the mouse melanoma B16-F1 cell line. **(A)** Immunofluorescence using antibodies targeting ARPC2 (a subunit of the Arp2/3 complex) or cortactin (marker of branched actin), phalloidin (probe targeting filamentous actin) and DAPI (marker of nuclei). Cells were treated for 1 h with 100 μM of the inactive control CK-689, CK-666 and its analog #59. Scale bar: 20 μm. **(B)** Quantification of cells displaying lamellipodia. For each compound, more than 120 cells from seven fields of view (n = 7) were analyzed. Mean ± S.D. Kruskal–Wallis test. Two biological replicates with three technical repeats were performed and gave similar results. Only one is displayed. *p < 0.05, **p < 0.01, ***p < 0.001, ****p < 0.0001, ns non-significant. **(C)** Viability of cells treated with 100 μM of compound for 4 h was measured using MTS (3-(4,5-dimethylthiazol-2-yl)-5-(3-carboxymethoxyphenyl)-2-(4-sulfophenyl)-2H-tetrazolium)). Mean ± S.D. of a single experiment with three technical repeats (n = 3). Kruskal–Wallis test. *p < 0.05, **p < 0.01, ***p < 0.001, ****p < 0.0001, ns non-significant. **(D)** Dose-dependent lamellipodium formation. The four selected compounds, **59**, **69**, **15** and **71** were compared to the active CK-666 and the inactive CK-689 compounds. For each compound, more than 130 cells from seven fields of view (n = 7) were analyzed per condition. Mean ± S.D. of a single experiment. *p*-values are given in the Supplementary Material (Supplementary Table S2). **(E)** Wound healing. CK-689 and CK-666 were at 100 μM, the four compounds **59**, **69**, **15** and **71** were at 25 μM. Nine wounded fields (n = 9) were imaged per condition and healing was measured over time. Mean ± S.D. is plotted for each time point. Several experiments were performed with different concentrations of analogs (25, 50 and 100 μM) in comparison with 100 μM of CK-666. One representative experiment with 25 μM of compounds is shown. *p*-values are given in the Supplementary Material (Supplementary Table S3). Plots of **15** and **69** are hardly visible, because they are superimposed to the plot of CK-666.

Arp2/3 activity is most established to generate membrane protrusions, called lamellipodia, that mediate a type of cell motility, referred to as mesenchymal. Thus, the next step was to evaluate whether the nine selected compounds inhibited lamellipodium formation and cell migration. These assays are more demanding than monitoring cell cycle progression by EdU incorporation. B16-F1 murine melanoma cells, which are well known to form prominent lamellipodia, were chosen. These lamellipodia were further enhanced by treating cells with aluminium fluoride (Hahne et al., 2001). Control B16-F1 cells treated with 100 μM of the inactive CK-689 compound formed a single wide lamellipodium encompassing most cell perimeter. As expected, these lamellipodia contained polymerized actin, visualized by phalloidin staining, Arp2/3 complexes, detected by immunofluorescence of the ARPC2 subunit, and cortactin that stabilizes branched actin junctions (Figure 2A) (Gautreau et al., 2021). CK-666 at 100 μM concentration blocked lamellipodium formation almost completely (Figure 2B). All nine selected compounds also efficiently inhibited lamellipodium formation. Six of them, **15, 52, 59, 64, 69, 71**, showed similar or higher activity compared to CK-666. Compounds **12**, **30** and **24**, were significantly less active than CK-666 (outside of the 1 S.D. range) and were excluded from further analysis. Compounds **52** and **64** were significantly toxic to B16-F1 cells, since they reduced the cell population by half or more (Figure 2C). They were therefore also excluded. The remaining four compounds, were evaluated in dose-dependent assays on lamellipodium formation (Figure 2D). At least two of them, compounds **15** and **71**, were significantly more potent than CK-666, whereas compound **69** was similar to CK-666, and compound **59** was slightly less active than CK-666.

For direct assessment of cell migration, we used a wound healing assay, where B16-F1 cells migrate to a cell-free zone obtained by scratching cells out with a pipette tip. This assay was less sensitive than the direct counting of cells displaying lamellipodia, but it allowed to directly observe cell migration over time. At 100 µM concentration, compounds **71**, **15**, and to a lesser extent **59** induced cell rounding followed by detachment from the substrate (Supplementary Video S1). This effect was not observed for **15** and **59** at 25 µM concentration (Supplementary Video S2). Compounds **15** and **69** at 25 µM concentration, inhibited cell migration as efficiently as 100 µM CK-666 (Figure 2E). Compound **59** at 25 µM appeared more active than CK-666 at 100 µM and compound **71** at 25 µM concentration inhibited cell migration even more effectively, but still induced cell rounding at this concentration.

### Effect of Selected Compounds on *in Vitro* Actin Polymerization

**FIGURE 3:**
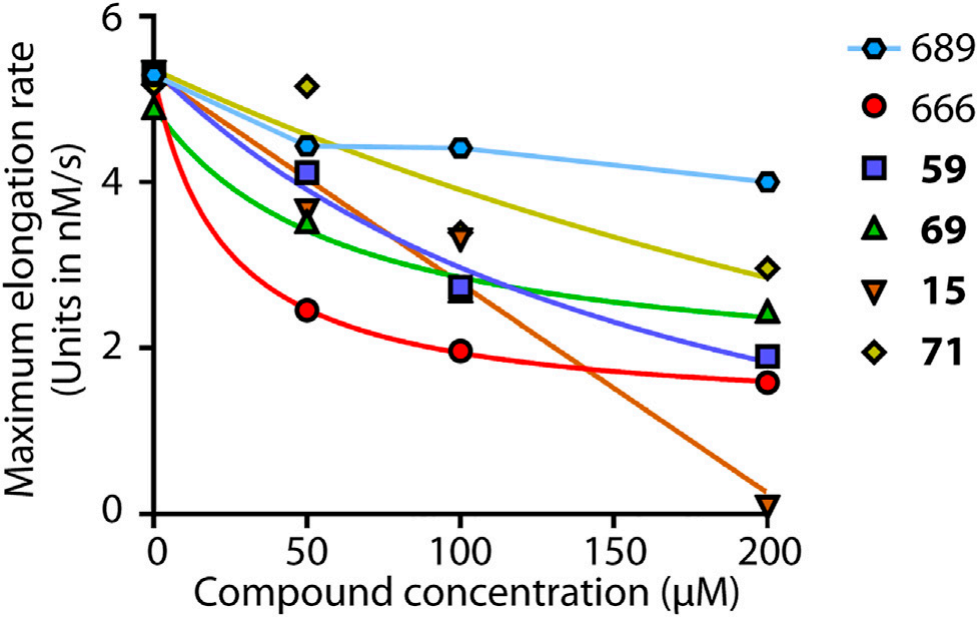
Arp2/3 inhibition *in vitro*. Four selected compounds, **59**, **69**, **15** and **71,** were compared to the active CK-666 and its inactive analog, CK-689. Polymerization of 1.5 μM actin (10% pyrenyl-labeled) was induced by 20 nM Arp2/3 and 250 nM N-WASP VCA and monitored by fluorescence increase of pyrenyl-labeled actin. The maximum polymerization rate was converted in nM/s. One representative experiment is shown, another experiment with slightly different conditions gave similar results. Compounds were dissolved in DMSO and total amount of DMSO was kept constant in the different conditions. The inactive inhibitor curve is not fitted.

We next addressed whether compounds **15**, **59**, **69**, and **71** inhibited Arp2/3 activity *in vitro*, as suggested by their effect on cell cycle progression and cell migration. To this end, we performed the classical fluorescence assay that monitors polymerization of actin coupled to pyrene. CK-666, but not CK-689, inhibited Arp2/3-mediated actin polymerization in a dose-dependent manner (Figure 3), in agreement with previous studies (Nolen et al., 2009; Hetrick et al., 2013). CK-666 displayed an IC_50_ of 23 μM and reached 78% of Arp2/3 inhibition at saturation. In this *in vitro* assay, the CK-666 analogs were overall less active than CK-666 itself. Compound **69** displayed an IC_50_ of 61 μM and reached 67% inhibition at saturation. IC_50_ of compounds **59** and **71** were 180 and 539 μM, respectively, and saturation was not reached. Compound **15** reached 100% inhibition in a linear manner, unlike CK-666 and the other analogs. Its IC_50_ was nonetheless 104 μM, higher than that of CK-666. Thus all four selected compounds were active against Arp2/3 *in vitro* although with different kinetic parameters than those of CK-666.

### *In Vivo* Effects of Selected Compounds on the Development of Sea Urchin Embryos

**FIGURE 4:**
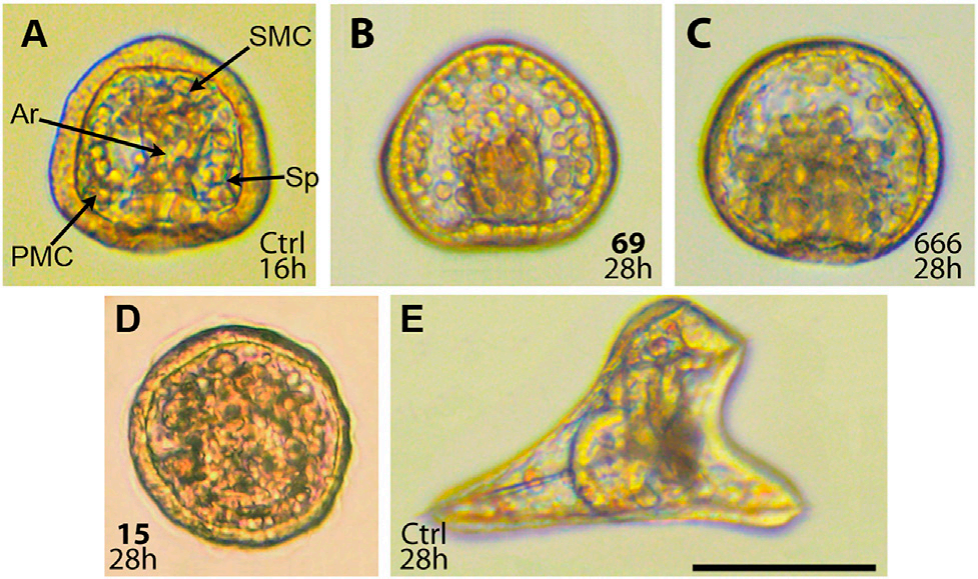
Effect of CK-666 analogs on sea urchin embryos. Embryos were treated with compounds at zygote stage (8–15 min post fertilization) and incubated at 22°C for the indicated time. **(A)** Untreated gastrula. Primary mesenchyme cells (PMC) form two ventrolateral clusters with triradiate spicule rudiments (Sp). Secondary mesenchyme cells (SMC) at the tip of archenteron (Ar) participate to archenteron elongation. **(B)** Defective gastrulation induced by compound **69** at 12 μM. **(C)** Defective gastrulation induced by CK-666 at 100 μM. Note altered distribution of primary and secondary mesenchyme cells, lack of spicules, and half-size archenteron. **(D)** Non-specific defects induced by compound **15** at 20 μM. Note the lack of obvious structures and low transparency. **(E)** Untreated larva at the early pluteus stage. Scale bar 100 μm.

The effects of compounds **15**, **59**, **69**, and **71** and standard Arp2/3 inhibitor CK-666 were examined using the sea urchin embryo model. Due to relatively large size and optical transparency of the embryos, this model allows for the direct monitoring of embryonic cell migration. Sea urchin gastrulation consists of two phases (Ettensohn, 1985). During the first one, primary mesenchyme cells (PMC) ingress into blastocoel in the vegetal pole and migrate to form ventrolateral clusters, where rudimental triradiate spicules appear (Figure 4). The second phase begins when the primary gut, the archenteron, reaches approximately its half-size. The secondary mesenchyme cells (SMC) emerging from the tip of archenteron pull the archenteron to the region of the future mouth.

According to literature, CK-666 at 100 μM concentration alters proper migration of PMC and formation of ventrolateral clusters with ensuing inhibition of spiculogenesis (Sepúlveda-ramírez et al., 2018). We studied the effect of CK-666 and its analogs **15**, **59**, **69**, and **71** over a longer period, up to the four-arm mid-pluteus stage. Compounds **59** and **69** as well as CK-666 altered PMC migration resulting in the lack of ventrolateral clusters and spicules. In addition, they inhibited elongation of the archenteron at approximately half-size (Figure 4), suggesting a developmental blockade at the transition from the first to the second phase of gastrulation, probably because of SMC defective migration. However, malformed gastrulae remained alive and were able to swim up to 34–35 h post fertilization. Compounds **59** and **69** were more active than CK-666 in this assay, inducing the same morphogenic aberrations at roughly 5-fold lower concentrations (Table 2).

**TABLE 2:** Effects of CK-666 and its analogs **15**, **59**, **69**, and **71** on sea urchin embryos.

Compound ID	Minimum effective concentration, μM		
	Systemic Toxicity	Inhibition of Spicule formation	Inhibition of Archenteron elongation
15	5	na	na
59	na	5	10
69	na	5	5
71	5	na	na
CK-666	na	25	50

Two biological repeats showed no difference in values of minimum effective concentrations.

Na: not applicable.

It should be noted that these compounds as wells as CK-666 up to 20–50 μM concentration did not affect cleavage and did not induce embryo spinning, typical effects of antimitotic microtubule destabilizing agents (Semenova et al., 2018). Beyond doubt, sea urchin embryo developmental alterations caused by CK-666 and the compound **59** and **69** were not caused by their side effect on another cytoskeletal system responsible for cell migration.

In contrast, compounds **15** and **71** induced nonspecific developmental abnormalities that were interpreted as systemic toxicity. At 5 μM concentration they suppressed plutei growth, whereas at 20 μM defective gastrulation was observed (Figure 4D). Due to low transparency of such gastrulae it was impossible to assess specific inhibitory effects related to PMC and SMC migration, such as inhibition of spicule formation and archenteron elongation.

In conclusion, *in vivo*, compounds **59** and **69** are Arp2/3 inhibitors with high potency and low toxicity.

### Docking of Active Compounds 59 and 69 Into the Arp2/3 Complex

Compounds **59** and **69** contain substituents in positions underexplored in previous studies (Nolen et al., 2009; Baggett et al., 2012; Hetrick et al., 2013). Therefore, to give flexibility to protein backbone and side chains, we used induced-fit docking (IFD) in the CK-666 binding site to allow ligand to adjust and optimize binding interactions with the Arp2/3 complex. The CK-666 binding site is at the interface between Arp2 and Arp3 subunits and CK-666 traps Arp2/3 in an inactive conformation (Nolen et al., 2009).

**FIGURE 5:**
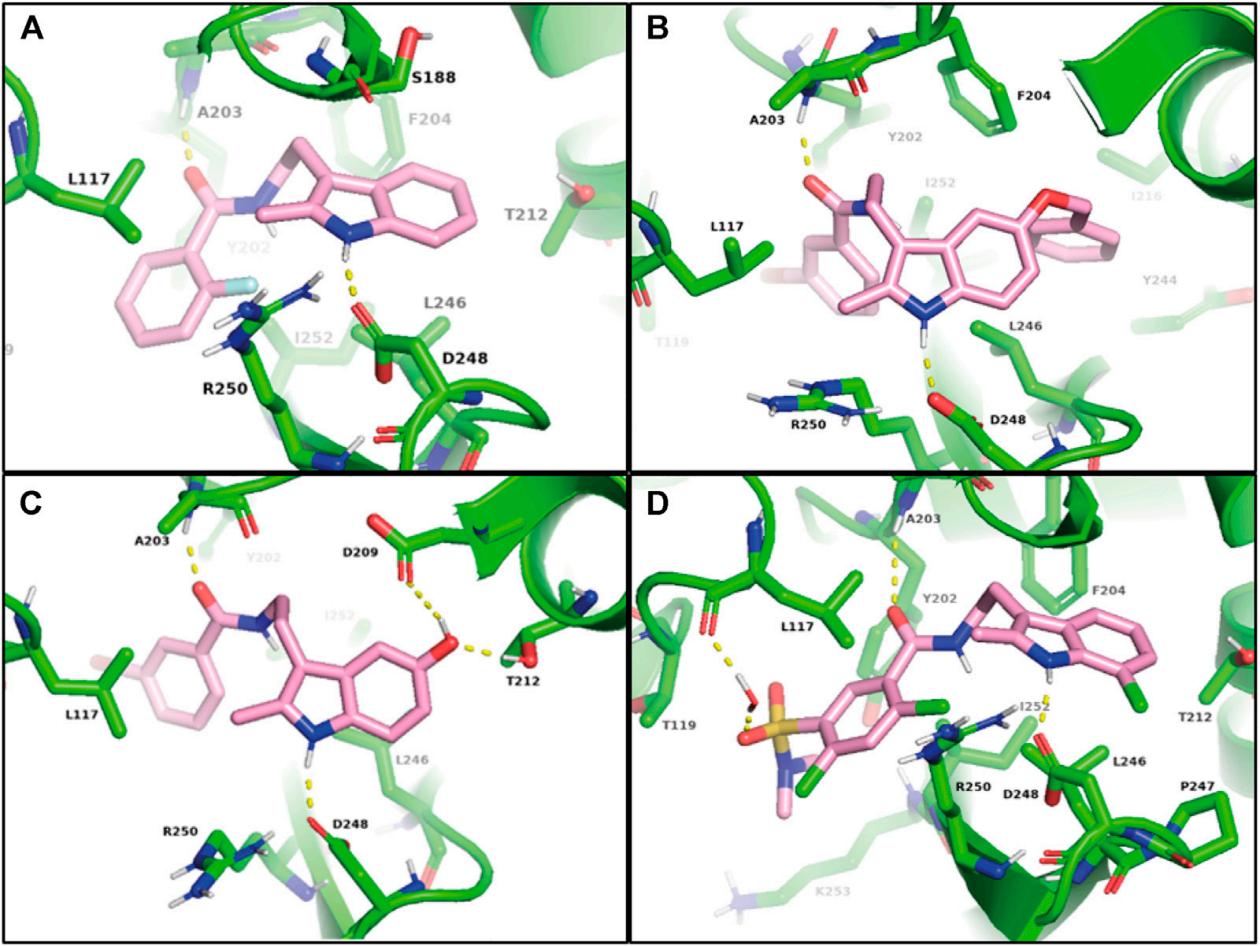
Models of Arp2/3 complex bound to CK-666 **(A)**, compound **59**, **(B)**, debenzylated metabolite of **59 (C)**, and compound **69 (D)**. Induced-fit docking was used to model binding of CK-666 analogs to the structure obtained by co-crystallizing CK-666 with the Arp2/3 complex (PDB code 3UKR).

As a control, we first applied the IFD procedure to CK-666 itself. We obtained a docking score of -7.644. The indole NH of CK-666 formed a hydrogen bound with the side chain of the conserved Asp248^Arp2^ and the amide oxygen of the CK-666 linker forms a hydrogen bound to the backbone NH of Ala203^Arp2^ (Figure 5A). The indole ring formed CH-π interactions with Ser188^Arp3^ and Leu246^Arp2^. The C2-methyl group bound to Leu117^Arp3^ and the aliphatic portion of Arg250^Arp2^. In addition, o-fluorobenzene ring filled the hydrophobic pocket created by Leu117^Arp3^, Thr119^Arp3^, Tyr202^Arp2^, Leu246^Arp2^, and Ile252^Arp2^, thus forming favorable interactions with the complex. All these predicted interactions of CK-666 were indeed observed in the co-crystal structure (Nolen et al., 2009).

Then the IFD procedure was applied to compound **59**. The best IFD results engaged benzoyl tryptamine of **59** in similar interactions to that observed with CK-666 bound Arp2/3 complex. It is associated with a docking score of -11.473. The bulky benzyl group of **59** induced a slight movement of β12 and β13 strands of Arp2 to occupy the lipophilic pocket formed by Phe204^Arp2^, Val213^Arp2^, Ile216^Arp2^, Tyr244^Arp2^, Leu246^Arp2^, and Ile252^Arp2^ (Figure 5B). The ortho-fluoro group of CK-666 formed an intramolecular hydrogen bond with the NH of its amide group. This locked the benzene ring of CK-666 in a conformation required for its inhibitory activity. In contrast, the meta-bromine atom of **59** did not favor this conformation. Compound **59**, however, can probably be debenzylated *in vivo* (Supplementary Scheme S1). Thus we performed IFD with the resulting metabolite, which provides a docking score of -8.361. The debenzylation yielded the C5 phenol hydroxyl group that might form two hydrogen bonds with Asp209^Arp2^ and Thr212^Arp2^ (Figure 5C).

The IFD of **69** gave a docking score of -7.975. The C7-chlorine group moved a loop between β12 and β13 strands of Arp2 and formed weak interactions with Pro247^Arp2^. A water molecule mediated contact between sulfonamide oxygen and the backbone C=O of Leu117^Arp3^. N-methyl of tertiary sulfonamide bound to Tyr202^Arp2^, Ile252^Arp2^, and Lys253^Arp2^ (Figure 5D). In conclusion, the best hits, **59** and **69**, probably bound to the same binding site as CK-666, but with significant variations.

## Discussion

Here we screened 74 structural analogs of CK-666 in a EdU incorporation assay. The role of the Arp2/3 complex in allowing cell cycle progression has only recently been described (Molinie et al., 2019) and is not yet as established as its role in lamellipodium protrusion and cell migration. Cell cycle progression, however, provided an easy read-out amenable to automated microscopy. This primary screen revealed nine active compounds, which all scored positive in secondary screens of lamellipodium formation and cell migration. EdU incorporation is thus a robust assay of Arp2/3 activity.

The relative potency of compounds depends on the assay. In the EdU assay, all nine hits displayed a decreased IC50 compared to CK-666, the reference compound. Yet this was not the case in lamellipodium formation and wound healing. Moreover, two compounds were excluded due to their toxicity in these cell assays, two others were excluded later on as a result of their toxicity in embryonic development of sea urchins.

The sea urchin embryo is a suitable model for compound screening (Semenova et al., 2008, 2018; Lichitsky et al., 2019). The phenotype associated with CK-666 mediated Arp2/3 inhibition (Sepúlveda-ramírez et al., 2018) was confirmed here with two other Arp2/3 inhibitory compounds. In addition to blocking migration of primary or secondary mesenchymal cells, Arp2/3 inhibition prevented secretion of triradiate spicules and archenteron elongation, which are straightforward to score. In this organism, our remaining best hits, **59** and **69**, were active at 5 to 10-fold lower concentrations than CK-666.

Our best hits were thus better Arp2/3 inhibitors than CK-666 *in vivo*, but performed less well *in vitro* in the classical assay of pyrene-actin polymerization. This was likely due to the *in vitro* selection scheme that yielded CK-666, whereas we used an *in vivo* selection scheme for the here studied analogs. Along this line, it must be noted that in the original publication reporting Arp2/3 inhibitors, CK-666 was found to be unable to inhibit the migration of fish keratocytes (Nolen et al., 2009), even if there is little doubt that it is an Arp2/3 dependent process (Dang et al., 2013; Koestler et al., 2013). CK-666 has now been shown to block lamellipodium-based migration in a variety of cell systems. Our compounds **59** and **69** might represent better alternatives to use *in vivo*.

The question as to why our best hits were less potent than CK-666 *in vitro* remains. One possibility is that different compounds do not inhibit Arp2/3 from different species with the same efficiency. In our assays, Arp2/3 was from human or mouse species for cell cultures, bovine for pyrene-actin assays and sea urchin embryo for the *in vivo* assay. We cannot rule out this hypothesis, but we do not favor it, since most Arp2/3 subunits are very highly conserved in mammals. We also verified that most key residues involved in compound binding upon *in silico* docking are conserved in sea urchins, with the exceptions of Phe204^Arp2^ and Ile216^Arp2^. An alternative explanation might be that our compounds better cross membranes for example, and would be more available to cells. This is certainly a possibility even if chemical structures of analogs do not obviously explain why. A last hypothesis, which is likely, is that cellular metabolism of compounds plays a role.

Compound-metabolizing enzymes cytochromes P450 are well expressed in the liver. Some of the cytochrome P450 enzymes, however, are widely expressed in embryonic and adult tissues (Yang et al., 1988; Iscan et al., 2001; Swanson, 2004). The here-proposed o-debenzylation, of **59** could be catalyzed by a cytochrome P450 or even occur spontaneously (Yang et al., 1988). It yields a compound of increased affinity based on our docking simulations. **69** is also likely to be metabolized *in vivo* by several pathways (García-Galán et al., 2008), one of them being the classical desulfonylation catalyzed by Glutathione-S-transferase *in vivo* (Bateman et al., 2010).

Compounds **59** and **69** display Arp2/3 binding modes almost similar to that of CK-666, but with some variations. The main electrostatic interactions mediating the affinity of CK-666 for the Arp2/3 are preserved in **59** and **69**. Substitutions that these compounds bear provide additional interactions and are likely to deform the Arp2/3 structure. A limitation of our study is that we did not prove that these synthesized compounds were indeed the ones inhibiting Arp2/3 *in vivo*. A future direction is thus to examine the possible metabolism of our compounds using mass spectrometry and the detailed binding modes of the resulting metabolites to the Arp2/3 by X-ray crystallography.

Our data show that **59** and **69** compounds featuring enhanced Arp2/3 inhibitory activity *in vivo* can be used in cell and developmental biology. Drugs that target actin polymerization are not yet used in the clinic for anticancer therapy, unlike drugs targeting microtubule dynamics, which are routinely used in cancer patients for their anti-proliferative activity. The actin cytoskeleton can also be a promising target for anticancer therapy (Stehn et al., 2013; Foerster et al., 2014). Synthetic lethality might be achieved by simultaneous application of inhibitors that target both cytoskeletal systems (Wang et al., 2020). The Arp2/3 inhibitor CDDO (To et al., 2010) suppressed tumor growth in mouse models of breast and lung cancer (Liby et al., 2007; Kim et al., 2012). Similarly, the two Arp2/3 inhibitors found through drug repositioning were also shown to block lung metastasis formed by pancreatic tumor cells in mouse models (Choi et al., 2019; Jin et al., 2019). Given the importance of Arp2/3 in cell migration and metastasis formation, evidenced by the association of Arp2/3 with high grade tumors and poor prognosis for patients (Molinie and Gautreau, 2018), the Arp2/3 complex is a target that should be seriously considered in the future for cancer therapy.

## Data Availability

The original contributions presented in the study are included in the article/Supplementary Material, further inquiries can be directed to the corresponding authors.
