# Supplementary material for: Synthesis, Screening and Characterization of Novel Potent Arp2/3 Inhibitory Compounds Analogous to CK-666
Source: Front Pharmacol. 2022 May 30;13:896994. doi: 10.3389/fphar.2022.896994 (PMC9189929; doi:10.3389/fphar.2022.896994)
Supplement: Supplementary file 3 [file DataSheet1.docx]

**Supplementary data**

**Synthesis, screening and characterization of novel potent Arp2/3 inhibitory compounds analogous to CK-666**

Artem I. Fokin^1,^*, Roman N. Chuprov-Netochin^2,†^, Alexander S. Malyshev^3,4,†^,

Stéphane Romero^1,†^, Marina N. Semenova^5,†^, Leonid D. Konyushkin^6^, Sergey V. Leonov^2^,

Victor V. Semenov^6^, Alexis M. Gautreau^1,7^*

^1^ CNRS UMR7654, Ecole Polytechnique, Institut Polytechnique de Paris, 91120 Palaiseau, France.

^2^ [Department of Molecular and Bio Physics](https://www.researchgate.net/institution/Moscow-Institute-of-Physics-and-Technology/department/Department-of-Molecular-and-Bio-Physics), [Moscow Institute of Physics and Technology](https://www.researchgate.net/institution/Moscow-Institute-of-Physics-and-Technology), Moscow Region, 141701, Russian Federation.

^3^ Lomonosov Moscow State University, Faculty of Medicine, 117192, Moscow, Russian Federation.

^4^ Dukhov Research Institute of Automatics (VNIIA), 127055, Moscow, Russian Federation.

^5^ N. K. Koltzov Institute of Developmental Biology RAS, 26 Vavilov Street, 119334 Moscow, Russian Federation.

^6^ N. D. Zelinsky Institute of Organic Chemistry RAS, 47 Leninsky Prospect, 119991 Moscow, Russian Federation.

^7^ Center of Molecular and Cellular Biology, Skolkovo Institute of Science and Technology, 30 Bolshoy Boulevard, 12105 Moscow, Russian Federation.

AIF present address:CNRS UMR168, Institut Curie, 75005 Paris, France.

* Correspondence should be addressed to AIF: [artem.fokin@curie.fr](mailto:artem.fokin@curie.fr)

and to AMG: [alexis.gautreau@polytechnique.edu](mailto:alexis.gautreau@polytechnique.edu)

† These authors, listed by alphabetic order, have contributed equally to this work.

**Table of Contents**

1) Video legends.  Page S3

2) Supplementary scheme S1. Possible metabolism of compound **59.** Page S4

3) Table S1. Structures of CK-666, and its analogues **1**–**74**. Pages S5-S8

4) Table S2. Supplementary data to **Figure 2D.** Page S9

5) Table S3. Supplementary data to **Figure 2E.** Page S10

6) Analytical data for CK666 and compounds **1-74.** Pages S11–S37

**Video legends**

**Video 1**

Wound healing assay of B16-F1 cells treated with 100 μM of CK-689, CK-666 and its analogs **59**, **69**, **15** and **71**. CK-666, but not CK-689, inhibits wound healing. Analogs **59**, **15** and **71** cause cell rounding and detachment. Scale bar: 100 μm. Total movie duration: 18 hours.

**Video 2**

Wound healing assay of B16-F1 cells treated with 100 μM of CK689, CK666 and 25 μM of its analogues **59**, **69**, **15** and **71**. The effect of analogs at 25 μM is comparable with 100 μM of CK666. Scale bar: 100 μm. Total movie duration: 18 hours.

**Supplementary schemes**


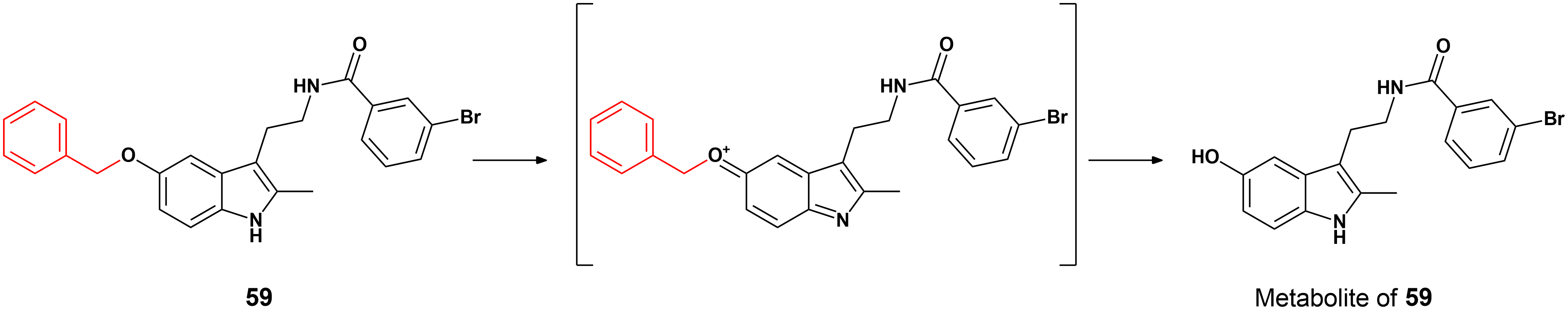


**Scheme S1**. Possible metabolism of compound **59**.

**Table S1**

Structures of CK-666, and its analogues **1**–**74**.

| Compound ID | Substitution | |
| --- | --- | --- |
|  | R | Ar |
| **CK-666** | H | 2-F-C_6_H_4_ |
| **1** | H | 4-F-C_6_H_4_ |
| **2** | H | 2-Br-C_6_H_4_ |
| **3** | H | 2-iPrO-C_6_H_4_ |
| **4** | H | 4-F_3_C-C_6_H_4_ |
| **5** | H | 2,6-diF-C_6_H_3_ |
| **6** | H | 2,6-diCl-C_6_H_3_ |
| **7** | H | 3,4-diCl-C_6_H_3_ |
| **8** | H | 2,4-diCl-C_6_H_3_ |
| **9** | H | 2-Cl-4,5-diF-C_6_H_2_ |
| **10** | H | 2-MeO-5-Cl-C_6_H_3_ |
| **11** | H | 2-NO_2_-C_6_H_4_ |
| **12** | H | 2-Cl-5-NO_2_-C_6_H_3_ |
| **13** | H | 3-NO_2_-4-Cl-C_6_H_3_ |
| **14** | H |  |
| **15** | H |  |
| **16** | H |  |
| **17** | H |  |
| **18** | H |  |
| **19** | H |  |
| **20** | H |  |
| **21** | 5-F_3_CO | 2-Thienyl |
| **22** | 5-Br | 2-Thienyl |
| **23** | 5-Me |  |
| **24** | 5-Me |  |
| **25** | 5-Me | 3-NO_2_-C_6_H_4_ |
| **26** | 5-Me | 4-Me-C_6_H_4_ |
| **27** | 5-Me | 2-Cl-4,5-diF-C_6_H_2_ |
| **28** | 5-Me |  |
| **29** | 5-Me |  |
| **30** | 5-Me |  |
| **31** | 5-Me |  |
| **32** | 5-MeO | 4-F-C_6_H_4_ |
| **33** | 5-MeO | 3,4-(MeO)_2_-C_6_H_3_ |
| **34** | 5-MeO | 3,4,5-(MeO)_3_-C_6_H_2_ |
| **35** | 5-MeO | 4-CO_2_Me-C_6_H_4_ |
| **36** | 5-MeO | 2-Cl-5-NO_2_-C_6_H_3_ |
| **37** | 5-MeO | 4-Me-3-NO_2_-C_6_H_3_ |
| **38** | 5-MeO |  |
| **39** | 5-MeO |  |
| **40** | 5-F_3_CO | 2-F-C_6_H_4_ |
| **41** | 5-F_3_CO | 2-Cl-C_6_H_4_ |
| **42** | 5-F_3_CO | 3,4-diF-C_6_H_3_ |
| **43** | 5-F_3_CO | 2-Cl-4,5-diF-C_6_H_2_ |
| **44** | 5-F_3_CO | 3-NO_2_-C_6_H_4_ |
| **45** | 5-F_3_CO | 2-Cl-5-NO_2_-C_6_H_3_ |
| **46** | 5-F_3_CO | 2-MeO-C_6_H_4_ |
| **47** | 5-F_3_CO | 4-MeO-C_6_H_4_ |
| **48** | 5-F_3_CO | 3,4-(MeO)_2_-C_6_H_3_ |
| **49** | 5-F_3_CO | 3,4,5-(MeO)_3_-C_6_H_2_ |
| **50** | 5-F_3_CO | 4-CO_2_Me-C_6_H_4_ |
| **51** | 5-F_3_CO |  |
| **52** | 5-F_3_CO |  |
| **53** | 5-F_3_CO | 5-Br-2-furyl |
| **54** | 5-F_3_CO |  |
| **55** | 5-F_3_CO |  |
| **56** | 5-BnO | 2-MeO-C_6_H_4_ |
| **57** | 5-BnO | 3,4-(MeO)_2_-C_6_H_3_ |
| **58** | 5-BnO | 4-CO_2_Me-C_6_H_4_ |
| **59** | 5-BnO | 3-Br-C_6_H_4_ |
| **60** | 5-F | 2,4-diCl-C_6_H_3_ |
| **61** | 5-F | 3-NO_2_-C_6_H_4_ |
| **62** | 5-F | 3,5-(NO_2_)_2_-C_6_H_3_ |
| **63** | 5-F |  |
| **64** | 5-F |  |
| **65** | 5-F |  |
| **66** | 5-F |  |
| **67** | 7-Cl | 3,5-(NO_2_)_2_-C_6_H_3_ |
| **68** | 7-Cl |  |
| **69** | 7-Cl |  |
| **70** | 7-F_3_C |  |
| **71** | 7-F_3_C |  |
| **72** | 4-F-7-Me | 4-CO_2_Me-C_6_H_4_ |
| **73** | 4-F-7-Me | 2-Me-C_6_H_4_ |
| **74** | 4-F-7-Me | 3,4-(MeO)_2_-C_6_H_3_ |

**Table S2. Supplementary data to Figure 2D.** Dose-dependent effect of Arp2/3 inhibitors on lamellipodia. P-values obtained in 2-way ANOVA analysis followed by pairwise Fisher's LSD (Least Significance Difference) test comparisons.

|  | 40μM | | 80μM | | 120μM | | 160μM | |
| --- | --- | --- | --- | --- | --- | --- | --- | --- |
|  | CK-689 | CK-666 | CK-689 | CK-666 | CK-689 | CK-666 | CK-689 | CK-666 |
| CK-689 | - | 0.0028  ** | - | <0.0001  **** | - | <0.0001  **** | - | <0.0001  **** |
| CK-666 | 0.0028  ** | - | <0.0001  **** | - | <0.0001  **** | - | <0.0001  **** | - |
| **59** | 0.0232  n.s. | 0.4627  n.s. | <0.0001  **** | 0.1543  n.s. | <0.0001  **** | 0.5149  n.s. | <0.0001  **** | 0.4205  n.s. |
| **69** | <0.0001  **** | 0.0016  ** | <0.0001  **** | 0.3933  n.s. | <0.0001  **** | 0.8957  n.s. | <0.0001  **** | 0.5468  n.s. |
| **15** | <0.0001  **** | <0.0001  **** | <0.0001  **** | 0.5097  n.s. | <0.0001  **** | 0.9986  n.s. | <0.0001  **** | 0.8260  n.s. |
| **71** | <0.0001  **** | 0.0001  **** | <0.0001  **** | 0.1456  n.s. | <0.0001  **** | 0.5928  n.s. | <0.0001  **** | 0.9837  n.s. |

**Table S3. Supplementary data to Figure 2E.** Effect of Arp2/3 inhibitors on cell migration. P-values obtained in 2-way ANOVA analysis followed by pairwise Fisher's LSD test comparisons.

|  | 6 hours | | 12 hours | | 18 hours | |
| --- | --- | --- | --- | --- | --- | --- |
|  | CK-689 | CK-666 | CK-689 | CK-666 | CK-689 | CK-666 |
| CK-689 | - | 0.0005  *** | - | <0.0001  **** | - | <0.0001  **** |
| CK-666 | 0.0005  *** | - | <0.0001  **** | - | <0.0001  **** | - |
| **59** | 0.8009  n.s. | 0.0406  * | <0.0001  **** | 0.4454  n.s. | <0.0001  **** | <0.0001  **** |
| **69** | 0.0002  *** | >0.9999  n.s. | <0.0001  **** | >0.9999  n.s. | <0.0001  **** | >0.9999  n.s. |
| **15** | 0.0014  ** | >0.9999  n.s. | <0.0001  **** | 0.9582  n.s. | <0.0001  **** | 0.3614  n.s. |
| **71** | 0.0076  ** | >0.9999  n.s. | <0.0001  **** | 0.0122  * | <0.0001  **** | <0.0001  **** |

**Analytical data for CK666 and compounds 1-74**

*2-Fluoro-N-[2-(2-methyl-1H-indol-3-yl)ethyl]benzamide, CK-666*

White crystals; yield 2.3 g (65%); mp 125–127 °C (*i*-PrOH); ^1^H NMR (DMSO-*d*_6_) *δ* 10.63 (1H, s, NH), 8.23 (1H, s, NHCO), 7.61 (1H, t, JH-H = 7.4 Hz, JH-F = 7.4 Hz, H-6'), 7.54-7.46 (3H, m, H-4,4',5'), 7.27 (1H, d, JH-H = 7.9 Hz, JH-F = 7.9 Hz, H-3'), 7.23 (1H, d, *J* = 7.8 Hz, H-7), 6.97 (1H, t, *J* = 7.4 Hz, H-6), 6.93 (1H, t, *J* = 7.4 Hz, H-5), 3.43 (2H, q, *J* = 6.8 Hz, CH2N), 2.90 (2H, t, *J* = 7.8 Hz, CH2), 2.34 (3H, s, CH3); EIMS *m*/*z* 297 [M+1]+ (6), 296 [M]+ (33), 158 (5), 157 (59), 145 (6), 144 (100), 123 (23), 95 (7). Anal. Calcd for C_18_H_17_FN_2_O: C 72.96; H 5.78; N 9.45%. Found: C 73.09; H 5.82; N 9.30%.

*4-Fluoro-N-[2-(2-methyl-1H-indol-3-yl)ethyl]benzamide (****1****)*

^1^H NMR (DMSO-*d*_6_) *δ* 10.62 (1H, s, NH), 8.51 (1H, s, NHCO), 7.90 (2H, dd, *J*_H-H_= 8.5 Hz, *J*_H-F_= 5.5 Hz,H-2',6'), 7.47 (1H, d, *J* = 7.7 Hz,H-4), 7.27 (2H, t, *J*_H-H_= 8.7 Hz, *J*_H-F_= 8.7 Hz,H-3',5'), 7.23 (1H, d, *J* = 7.9 Hz,H-7), 6.97 (1H, t, *J* = 7.5 Hz, H-6), 6.92 (1H, t, *J* = 7.5 Hz, H-5), 3.41 (2H, q, *J* = 7.0 Hz, CH_2_N), 2.89 (2H, t, *J* = 7.6 Hz, CH_2_), 2.32 (3H, s, CH_3_); EIMS *m*/*z 297* [M+1]^+^ (10), 296 [M]^+^ (45), 158 (14), 157 (100), 156 (5), 145 (15), 144 (99), 143 (22), 128 (5), 123 (30), 115 (7), 95 (20), 75 (6). Anal. Calcd for C_18_H_17_FN_2_O: C 72.96; H 5.78; N 9.45%. Found: C 73.05; H 5.80; N 9.38%.

*2-Bromo-N-[2-(2-methyl-1H-indol-3-yl)ethyl]benzamide (****2****)*

^1^H NMR (DMSO-*d*_6_) *δ* 10.57 (1H, s, NH), 8.30 (1H, s, NHCO), 7.61 (1H, d, *J* = 7.8 Hz,H-6'), 7.47 (1H, d, *J* = 7.6 Hz, H-4), 7.38 (1H, t, *J* = 7.4 Hz, H_arom_), 7.35-7.28 (2H, m, H_arom_), 7.22 (1H, d, *J* = 7.8 Hz, H-7), 6.97 (1H, t, *J* = 7.8 Hz, H-6), 6.92 (1H, t, *J* = 7.3 Hz, H-5), 3.39 (2H, q, *J* = 7.0 Hz, CH_2_N), 2.90 (2H, t, *J* = 7.8 Hz, CH_2_), 2.38 (3H, s, CH_3_); EIMS *m*/*z 358* [M]^+^ (9), *356* [M]^+^ (9), 185 (18), 183 (19), 157 (87), 156 (16), 145 (33), 144 (100), 143 (57, 130 (10), 128 (11), 115 (18), 105 (10), 103 (10), 102 (10), 77 (24)). Anal. Calcd for C_18_H_17_BrN_2_O: C 60.52; H 4.80; N 7.84%. Found: C 60.59; H 4.83; N 7.76%.

*2-Isopropoxy-N-[2-(2-methyl-1H-indol-3-yl)ethyl]benzamide (****3****)*

^1^H NMR (DMSO-*d*_6_) * *10.74 (1H, s, NH), 8.14 (1H, t, *J* = 5.7 Hz,NHCO), 7.85 (1H, dd, *J* = 7.8 Hz, *J* = 1.8 Hz,H-6'), 7.47 (1H, d, *J* = 7.8 Hz,H-4), 7.42 (1H, t, *J* = 8.1 Hz,H-4'), 7.24 (1H, d, *J* = 8.0 Hz,H-7), 7.10 (1H, d, *J* = 8.3 Hz, *H-3*'), 7.01 (1H, t, *J* = 7.5 Hz, H-5'), 6.98 (1H, t, *J* = 7.3 Hz,H-6), 6.91 (1H, t, *J* = 7.3 Hz,H-5), 4.68-4.61 (1H, m, CHO), 3.52 (2H, q, *J* = 6.8 Hz, CH_2_N), 2.88 (2H, t, *J* = 7.2 Hz, CH_2_), 2.30 (3H, s, CH_3_-2), 1.10 (3H, s, CH_3_), 1.09 (3H, s, CH_3_); EIMS *m*/*z* 336 [M]^+^ (3), 158 (10), 157 (100), 145 (6), 144 (56), 143 (19), 138 (8), 121 (18), 120 (5), 115 (5), 77 (6). Anal. Calcd for C_21_H_24_N_2_O_2_: C 74.97; H 7.19; N 8.33%. Found: C 75.12 H 7.23; N 8.20%.

*N-[2-(2-Methyl-1H-indol-3-yl)ethyl]-4-(trifluoromethyl)benzamide (****4****)*

^1^H NMR (DMSO-*d*_6_) *δ* 10.56 (1H, s, NH), 8.67 (1H, s, NHCO), 8.02 (2H, d, *J* = 8.0 Hz,H-2',6'), 7.78 (2H, d, *J* = 8.0 Hz, H-3',5'), 7.46 (1H, d, *J* = 7.6 Hz, H-4), 7.22 (1H, d, *J* = 7.9 Hz, H-7), 6.96 (1H, t, *J* = 7.3 Hz, H-6), 6.91 (1H, t, *J* = 7.3 Hz, H-5), 3.45 (2H, q, *J* = 7.1 Hz, CH_2_N), 2.92 (2H, t, *J* = 7.5 Hz, CH_2_), 2.34 (3H, s, CH_3_); EIMS *m*/*z 346* [M]^+^ (4), 173 (11), 158 (6), 157 (50), 145 (24), 144 (100), 143 (18), 115 (6), 77 (5). Anal. Calcd for C_19_H_17_ F_3_N_2_O: C 65.89; H 4.95; N 8.09%. Found: C 66.02; H 4.98; N 7.96%.

*2,6-Difluoro-N-[2-(2-methyl-1H-indol-3-yl)ethyl]benzamide (****5****)*

^1^H NMR (DMSO-*d*_6_) *δ* 10.58 (1H, s, NH), 8.62 (1H, s, NHCO), 7.51-7.43 (2H, m, H-4,4'), 7.22 (1H, d, *J* = 7.7 Hz,H-7), 7.09 (2H, t, *J*_H-H_= 7.9 Hz, *J*_H-F_= 7.9 Hz,H-3', 5'), 6.96 (1H, t, *J* = 7.0 Hz, H-6), 6.92 (1H, t, *J* = 7.0 Hz, H-5), 3.41 (2H, q, *J* = 7.0 Hz, CH_2_N), 2.89 (2H, t, *J* = 7.7 Hz, CH_2_), 2.36 (3H, s, CH_3_); EIMS *m*/*z 315* [M+1]^+^ (6), *314* [M]^+^ (30), 158 (9), 157 (72), 145 (12), 144 (100), 143 (14), 141 (23), 113 (7). Anal. Calcd for C_18_H_16_F_2_N_2_O: C 68.78; H 5.13; N 8.91%. Found: C 68.86; H 5.15; N 8.86%.

*2,6-Dichloro-N-[2-(2-methyl-1H-indol-3-yl)ethyl]benzamide (****6****)*

^1^H NMR (DMSO-*d*_6_) *δ* 10.67 (1H, s, NH), 8.70 (1H, s, NHCO), 7.52-7.46 (3H, m, H-3',4',5'), 7.42 (1H, dd, *J* = 9.0 Hz, *J* = 7.1 Hz, H-4), 7.23 (1H, d, *J* = 7.8 Hz, H-7), 6.99 (1H, t, *J* = 7.3 Hz, H-6), 6.95 (1H, t, *J* = 7.3 Hz, H-5), 3.45-3.39 (2H, m, CH_2_N), 2.90 (2H, t, *J* = 7.5 Hz, CH_2_), 2.36 (3H, s, CH_3_); EIMS *m*/*z 346* [M]^+^ (1), 175 (16), 157 (50), 147 (10), 145 (22), 144 (100), 143 (32), 115 (13), 109 (11), 77 (12). Anal. Calcd for C_18_H_16_ Cl_2_N_2_O: C 62.26; H 4.64; N 8.07%. Found: C 62.36; H 4.67; N 8.00%.

*3,4-Dichloro-N-[2-(2-methyl-1H-indol-3-yl)ethyl]benzamide (****7****)*

^1^H NMR (DMSO-*d*_6_) *δ* 10.63 (1H, s, NH), 8.70 (1H, s, NHCO), 8.04 (1H, d, *J* = 2.1 Hz,H-2'), 7.81 (1H, dd, *J* = 8.3 Hz, *J* = 2.1 Hz,H-6'), 7.73 (1H, d, *J* = 8.3 Hz,H-5'), 7.45 (1H, d, *J* = 7.8 Hz, H-4), 7.23 (1H, d, *J* = 7.3 Hz, H-7), 6.97 (1H, t, *J* = 7.3 Hz, H-6), 6.92 (1H, t, *J* = 7.3 Hz, H-5), 3.41 (2H, q, *J* = 6.9 Hz, CH_2_N), 2.90 (2H, t, *J* = 7.4 Hz, CH_2_), 2.31 (3H, s, CH_3_); EIMS *m*/*z 350* [M]^+^ (2), *348* [M]^+^ (9), *346* [M]^+^ (14), 175 (12), 173 (17), 157 (48), 147 (7), 145 (14), 144 (100). Anal. Calcd for C_18_H_16_Cl_2_N_2_O: C 62.26; H 4.64; N 8.07%. Found: C 62.37; H 4.67; N 7.94%.

*2,4-Dichloro-N-[2-(2-methyl-1H-indol-3-yl)ethyl]benzamide (****8****)*

^1^H NMR (DMSO-*d*_6_) *δ* 10.57 (1H, s, NH), 8.37 (1H, s, NHCO), 7.56 (1H, d, *J* = 2.1 Hz,H-3'), 7.46 (1H, d, *J* = 7.7 Hz, H-6'), 7.41 (1H, dd, *J* = 8.2 Hz, *J* = 2.1 Hz, H-5'), 7.35 (1H, d, *J* = 8.1 Hz, H-4), 7.22 (1H, d, *J* = 7.9 Hz, H-7), 6.96 (1H, t, *J* = 7.2 Hz, H-6), 6.92 (1H, t, *J* = 7.2 Hz, H-5), 3.39 (2H, q, *J* = 7.0 Hz, CH_2_N), 2.90 (2H, t, *J* = 7.8 Hz, CH_2_), 2.38 (3H, s, CH_3_); EIMS *m*/*z 348* [M]^+^ (1), *346* [M]^+^ (2), 175 (6), 173 (10), 158 (8), 157 (65), 156 (6), 145 (22), 144 (100), 143 (17), 77 (5). Anal. Calcd for C_18_H_16_ Cl_2_N_2_O: C 62.26; H 4.64; N 8.07%. Found: C 62.39; H 4.68; N 7.96%.

*2-Chloro-4,5-difluoro-N-[2-(2-methyl-1H-indol-3-yl)ethyl]benzamide (****9****)*

^1^H NMR (DMSO-*d*_6_) *δ* 10.60 (1H, s, NH), 8.42 (1H, s, NHCO), 7.65 (1H, dd, *J* = 10.4 Hz, *J* = 7.0 Hz,H-6'), 7.46 (1H, d, *J* = 7.7 Hz,H-4), 7.36 (1H, dd, *J* = 10.4 Hz, *J* = 8.8 Hz,H-3'), 7.23 (1H, d, *J* = 7.8 Hz, H-7), 6.97 (1H, t, *J* = 7.2 Hz, H-6), 6.92 (1H, t, *J* = 7.2 Hz, H-5), 3.40 (2H, q, *J* = 7.0 Hz, CH_2_N), 2.90 (2H, t, *J* = 7.6 Hz, CH_2_), 2.37 (3H, s, CH_3_); EIMS *m*/*z 350* [M]^+^ (2), *348* [M]^+^ (6), 175 (6), 158 (6), 157 (52), 156 (6), 145 (12), 144 (100), 143 (16), 115 (5), 77 (6). Anal. Calcd for C_18_H_15_ClF_2_N_2_O: C 61.99; H 4.33; N 8.03%. Found: C 62.12; H 4.36; N 7.92%.

*5-Chloro-2-methoxy-N-[2-(2-methyl-1H-indol-3-yl)ethyl]benzamide (****10****)*

^1^H NMR (DMSO-*d*_6_) *δ* 10.69 (1H, s, NH), 8.15 (1H, s, NHCO), 7.72 (1H, d, *J* = 2.8 Hz,H-6'), 7.52-7.46 (2H, m, H-3',4'), 7.24 (1H, d, *J* = 9.0 Hz, H-4), 7.13 (1H, d, *J* = 8.8 Hz, H-7), 6.98 (1H, t, *J* = 7.5 Hz, H-6), 6.93 (1H, t, *J* = 7.5 Hz, H-5), 3.73 (3H, s, OCH_3_), 3.48 (2H, q, *J* = 6.8 Hz, CH_2_N), 2.90 (2H, t, *J* = 7.2 Hz, CH_2_), 2.34 (3H, s, CH_3_); EIMS *m*/*z 344* [M]^+^ (1), *342* [M]^+^ (3), 171 (8), 169 (26), 158 (8), 157 (72), 145 (11), 144 (100), 143 (27), 128 (11), 126 (15), 115 (10), 111 (14), 102 (5), 77 (13). Anal. Calcd for C_19_H_19_ClN_2_O_2_: C 66.57; H 5.59; N 8.17%. Found: C 66.64; H 5.61; N 8.12%.

*N-[2-(2-Methyl-1H-indol-3-yl)ethyl]-2-nitrobenzamide (****11****)*

^1^H NMR (DMSO-*d*_6_) *δ* 10.57 (1H, s, NH), 8.60 (1H, s, NHCO), 7.98 (1H, d, *J* = 9.0 Hz,H-3'), 7.72 (1H, t, *J* = 7.5 Hz, H-5'), 7.65 (1H, t, *J* = 7.7 Hz, H-4'), 7.49 (1H, d, *J* = 7.4 Hz, H-6'), 7.46 (1H, d, *J* = 7.6 Hz, H-4), 7.22 (1H, d, *J* = 7.8 Hz, H-7), 6.97 (1H, t, *J* = 7.5 Hz, H-6), 6.92 (1H, t, *J* = 7.3 Hz, H-5), 3.40 (2H, q, *J* = 7.0 Hz, CH_2_N), 2.90 (2H, t, *J* = 7.7 Hz, CH_2_), 2.38 (3H, s, CH_3_); EIMS *m*/*z 323* [M]^+^ (12), 158 (7), 157 (55), 145 (11), 144 (100), 143 (11). Anal. Calcd for C_18_H_17_N_3_O_3_: C 66.86; H 5.30; N 13.00%. Found: C 66.80; H 5.28; N 13.10%.

*2-Chloro-N-[2-(2-methyl-1H-indol-3-yl)ethyl]-5-nitrobenzamide (****12****)*

^1^H NMR (DMSO-*d*_6_) *δ* 10.38 (1H, s, NH), 8.39 (1H, s, NHCO), 8.20 (1H, dd, *J* = 8.6 Hz, *J* = 2.4 Hz,H-4'), 8.15 (1H, d, *J* = 2.4 Hz,H-6'), 7.67 (1H, d, *J* = 8.9 Hz,H-3'), 7.46 (1H, d, *J* = 7.6 Hz, H-4), 7.21 (1H, d, *J* = 7.6 Hz, H-7), 6.96-6.88 (2H, m, H-5,6), 3.49 (2H, q, *J* = 7.0 Hz, CH_2_N), 2.96 (2H, t, *J* = 7.3 Hz, CH_2_), 2.42 (3H, s, CH_3_); EIMS *m*/*z 357* [M]^+^ (1), 184 (12), 157 (28), 145 (11), 144 (100), 143 (44), 138 (16), 115 (13), 110 (13), 77 (14). Anal. Calcd for C_18_H_16_ClN_3_O_3_: C 60.42; H 4.51; N 11.74%. Found: C 60.37; H 4.48; N 11.84%.

*4-Chloro-N-[2-(2-methyl-1H-indol-3-yl)ethyl]-3-nitrobenzamide (****13****)*

^1^H NMR (DMSO-*d*_6_) *δ* 10.66 (1H, s, NH), 8.87 (1H, s, NHCO), 8.46 (1H, d, *J* = 2.1 Hz,H-2'), 8.12 (1H, dd, *J* = 8.3 Hz, *J* = 2.1 Hz,H-6'), 7.89 (1H, d, *J* = 8.3 Hz,H-5'), 7.45 (1H, d, *J* = 7.8 Hz, H-4), 7.23 (1H, d, *J* = 7.9 Hz, H-7), 6.96 (1H, t, *J* = 7.3 Hz, H-6), 6.92 (1H, t, *J* = 7.3 Hz, H-5), 3.44 (2H, q, *J* = 6.9 Hz, CH_2_N), 2.91 (2H, t, *J* = 7.4 Hz, CH_2_), 2.31 (3H, s, CH_3_); EIMS *m*/*z 359* [M]^+^ (3), *357* [M]^+^ (9), 184 (15), 157 (32), 145 (8), 144 (100), 138 (9). Anal. Calcd for C_18_H_16_ClN_3_O_3_: C 60.42; H 4.51; N 11.74%. Found: C 60.39; H 4.48; N 11.80%.

*N-(2-{[2-(2-Methyl-1H-indol-3-yl)ethyl]carbamoyl}phenyl)thiophene-2-carboxamide (****14****)*

^1^H NMR (DMSO-*d*_6_) *δ* 12.61 (1H, s, NHCO), 10.64 (1H, s, NH), 8.91 (1H, s, NHCO), 8.53 (1H, d, *J* = 8.3 Hz,H-3'), 7.90 (1H, d, *J* = 5.0 Hz, H-3''), 7.75 (1H, d, *J* = 7.9 Hz, H-6'), 7.72 (1H, d, *J* = 3.7 Hz, H-5''), 7.54 (1H, t, *J* = 7.9 Hz,H-5'), 7.48 (1H, d, *J* = 7.7 Hz, H-4), 7.27 (1H, t, *J* = 4.4 Hz, H-4''), 7.22 (1H, d, *J* = 7.9 Hz, H-7), 7.16 (1H, t, *J* = 7.6 Hz, H-4'), 6.97 (1H, t, *J* = 7.5 Hz, H-6), 6.92 (1H, t, *J* = 7.3 Hz, H-5), 3.50 (2H, q, *J* = 6.7 Hz, CH_2_N), 2.95 (2H, t, *J* = 7.2 Hz, CH_2_), 2.31 (3H, d, *J* = 4.0 Hz, CH_3_); EIMS *m*/*z 404* [M+1]^+^ (1), 403 [M]^+^ (3), 230 (6), 158 (13), 157 (100), 156 (7), 146 (8), 145 (11), 144 (79), 143 (12), 120 (6), 111 (24). Anal. Calcd for C_23_H_21_N_3_O_2_S: C 68.46; H 5.25; N 10.41%. Found: C 68.50; H 5.27; N 10.47%.

*2-Benzoyl-N-[2-(2-methyl-1H-indol-3-yl)ethyl]benzamide (****15****)*

^1^H NMR (DMSO-*d*_6_) *δ* 10.58 (1H, s, NH), 7.75 (1H, d, *J* = 7.0 Hz,H-6'), 7.61-7.50 (2H, m, NHCO, H-3'), 7.44 -7.24 (7H, m, H-4,H_arom_), 7.18 (1H, d, *J* = 7.9 Hz, H-7), 7.06 (1H, s, H_arom_), 6.94 (1H, t, *J* = 7.5 Hz, H-6), 6.88 (1H, t, *J* = 7.4 Hz, H-5), 3.40 (1H, td, *J* = 13.0 Hz, *J* = 5.0 Hz, CH_2_N), 3.12 (1H, td, *J* = 13.0 Hz, *J* = 5.0 Hz, CH_2_N), 2.93 (1H, td, *J* = 13.0 Hz, *J* = 4.7 Hz, CH_2_), 2.56 (1H, td, *J* = 13.0 Hz, *J* = 4.7 Hz, CH_2_), 2.22 (3H, s, CH_3_); EIMS *m*/*z 383* [M+1]^+^ (3), *382* [M]^+^ (11), 209 (24), 158 (11), 157 (80), 156 (6), 152 (10), 145 (12), 144 (100), 143 (14), 115 (5), 105 (6), 77 (12)). Anal. Calcd for C_25_H_22_N_2_O_2_: C 78.51; H 5.80; N 7.32%. Found: C 78.66; H 5.83; N 7.20%.

*N-[2-(2-Methyl-1H-indol-3-yl)ethyl]-4-(morpholin-4-ylsulfonyl)benzamide (****16****)*

^1^H NMR (DMSO-*d*_6_) *δ* 10.62 (1H, s, NH), 8.75 (1H, s, NHCO), 8.05 (2H, d, *J* = 8.1 Hz,H-2',6'), 7.82 (2H, d, *J* = 8.1 Hz, H-3',5'), 7.47 (1H, d, *J* = 7.7 Hz, H-4), 7.23 (1H, d, *J* = 7.9 Hz, H-7), 6.98 (1H, t, *J* = 7.5 Hz, H-6), 6.92 (1H, t, *J* = 7.3 Hz, H-5), 3.64 (4H, t, *J* = 4.4 Hz,H-2'',6''), 3.45 (2H, q, *J* = 6.9 Hz, CH_2_N), 2.92 (4H, m, H-3'',5''), 2.89 (2H, t, *J* = 7.5 Hz, CH_2_), 2.32 (3H, s, CH_3_); EIMS *m*/*z 427* [M]^+^ (2), 158 (7), 145 (9), 144 (100), 76 (5). Anal. Calcd for C_22_H_25_ N_3_O_4_S: C 61.81; H 5.89; N 9.83%. Found: C 61.89; H 5.93; N 9.74%.

*N-[2-(2-Methyl-1H-indol-3-yl)ethyl]-1-(phenylsulfonyl)prolinamide (****17****)*

^1^H NMR (DMSO-*d*_6_) *δ* 10.39 (1H, s, NH), 7.83 (2H, d, *J* = 7.8 Hz, H-2'',6''), 7.64 (1H, t, *J* = 7.4 Hz,H-4''), 7.62-7.54 (3H, m, NHCO, H-3'',5''), 7.44 (1H, d, *J* = 7.5 Hz, H-4), 7.20 (1H, d, *J* = 7.7 Hz, H-7), 6.97-6.87 (2H, m, H-5,6), 4.03 (1H, dd, *J* = 8.5 Hz, *J* = 3.3 Hz,H-2'), 3.40 (1H, m, H-5'), 3.32 (2H, m, CH_2_N), 3.17 (1H, q, *J* = 8.5 Hz, H-5'), 2.83 (3H, t, *J* = 7.4 Hz, CH_2_), 2.40 (3H, s, CH_3_), 1.88 (1H, m, H-3'), 1.73 (1H, m, H-3'), 1.63-148 (2H, m, H-4'); EIMS *m*/*z 411* [M]^+^ (9), 210 (36), 158 (16), 157 (100), 145 (6), 144 (98), 141 (21), 115 (5), 77 (56). Anal. Calcd for C_22_H_25_ N_3_O_3_S: C 64.21; H 6.12; N 10.21%. Found: C 64.31; H 6.16; N 10.11%.

*N^2^-[2-(2-Methyl-1H-indol-3-yl)ethyl]-N^1^-phenylpyrrolidine-1,2-dicarboxamide (****18****)*

^1^H NMR (DMSO-*d*_6_) *δ* 10.33 (1H, s, NH), 7.93 (1H, s, NHCO), 7.61 (1H, s, NHCO), 7.48(2H, d, *J* = 8.1 Hz, H-2'',6''), 7.42 (1H, d, *J* = 7.7 Hz, H-4), 7.22-7.15 (3H, m, H-7,H-3'',5''), 6.94-6.85 (3H, m, H-5,6, H-4''), 4.35 (1H, dd, *J* = 7.8 Hz, *J* = 3.2 Hz,H-2'), 3.59 (1H, m, H-5'), 3.45 (2H, m, CH_2_N), 3.28 (1H, m, H-5'), 2.80 (3H, t, *J* = 7.5 Hz, CH_2_), 2.37 (3H, s, CH_3_), 2.06-1.87 (4H, m, H-3',4'); EIMS *m*/*z 390* [M]^+^ (8), 189 (6), 158 (19), 157 (94), 145 (9), 144 (58), 143 (12), 119 (17), 115 (8), 91 (9), 77 (6), 71 (6), 70 (100). Anal. Calcd for C_23_H_26_ N_4_O_2_: C 70.75; H 6.71; N 14.35%. Found: C 70.84; H 6.76; N 14.26%.

*3-(4-Methoxyphenyl)-N-[2-(2-methyl-1H-indol-3-yl)ethyl]isoxazole-5-carboxamide (****19****)*

^1^H NMR (DMSO-*d*_6_) *δ* 10.74 (1H, s, NH), 9.07 (1H, t, *J* = 5.8 Hz, NHCO), 7.86 (2H, d, *J* = 8.8 Hz,H-2'',6''), 7.53 (1H, s, H-4'), 7.47 (1H, d, *J* = 7.6 Hz, H-4), 7.23 (1H, d, *J* = 7.8 Hz, H-7), 7.09 (2H, d, *J* = 8.8 Hz,H-3'',5''), 6.98 (1H, t, *J* = 7.1 Hz, H-6), 6.93 (1H, d, *J* = 7.1 Hz,H-5), 3.83 (3H, s, OCH_3_), 3.42 (2H, q, *J* = 7.0 Hz, CH_2_N), 2.91 (2H, t, *J* = 7.5 Hz, CH_2_), 2.32 (3H, s, CH_3_); EIMS *m*/*z 375* [M]^+^ (7), 174 (6), 157 (29), 146 (8), 145 (8), 144 (100). Anal. Calcd for C_22_H_21_N_3_O_3_: C 70.38; H 5.64; N 11.19%. Found: C 70.54; H 5.69; N 11.05%.

*4-Amino-N-[2-(2-methyl-1H-indol-3-yl)ethyl]-1,2,5-oxadiazole-3-carboxamide 2-oxide (****20****)*

^1^H NMR (DMSO-*d*_6_) *δ* 10.64 (1H, s, NH), 8.34 (1H, s, NHCO), 7.48 (1H, d, *J* = 7.8 Hz, H-4), 7.23 (1H, d, *J* = 8.0 Hz, H-7), 6.97 (1H, dt, *J* = 7.0 Hz, *J* = 1.1 Hz, H-6), 6.92 (1H, dt, *J* = 7.0 Hz, *J* = 1.1 Hz, H-5), 6.51 (2H, s, NH_2_), 3.48 (2H, q, *J* = 7.0 Hz, CH_2_N), 2.90 (2H, t, *J* = 7.5 Hz, CH_2_), 2.34 (3H, d, *J* = 1.8 Hz, CH_3_); EIMS *m*/*z 302* [M+1]^+^ (2), *301* [M]^+^ (11), 158 (22), 157 (13), 145 (11), 144 (100), 143 (19), 77 (5). Anal. Calcd for C_14_H_15_N_5_O_3_: C 55.81; H 5.02; N 23.24%. Found: C 55.76; H 4.98; N 23.31%.

*N-{2-[2-Methyl-5-(trifluoromethoxy)-1H-indol-3-yl]ethyl}thiophene-2-carboxamide (****21****)*

^1^H NMR (DMSO-*d*_6_) *δ* 10.96 (1H, s, NH), 8.50 (1H, s, NHCO), 7.69 (2H, dd, *J* = 21.2 Hz, *J* = 4.4 Hz,H-3',5'), 7.40 (1H, s, H-4), 7.30 (1H, d, *J* = 8.5 Hz, H-7), 7.12 (1H, t, *J* = 4.4 Hz,H-4'), 6.93 (1H, t, *J* = 8.3 Hz, H-6), 3.37 (2H, q, *J* = 6.9 Hz, CH_2_N), 2.88 (2H, t, *J* = 7.2 Hz, CH_2_), 2.33 (3H, s, CH_3_); EIMS *m*/*z 368* [M]^+^ (7), 242 (12), 241 (100), 229 (11), 228 (93), 158 (6), 131 (12), 130 (6), 111 (36). Anal. Calcd for C_17_H_15_F_3_N_2_O_2_S: C 55.43; H 4.10; N 7.60%. Found: C 55.39; H 4.08; N 7.56%.

*N-[2-(5-Bromo-2-methyl-1H-indol-3-yl)ethyl]thiophene-2-carboxamide (****22****)*

^1^H NMR (DMSO-*d*_6_) *δ* 10.57 (1H, s, NH), 8.15 (1H, s, NHCO), 7.61 (1H, d, *J* = 3.6 Hz,H-3'), 7.58 (1H, s, H-4), 7.49 (1H, d, *J* = 5.0 Hz, H-5'), 7.14 (1H, d, *J* = 8.4 Hz,H-7), 7.06-6.99 (2H, m, H-4',6), 3.40 (2H, q, *J* = 6.9 Hz, CH_2_N), 2.89 (2H, t, *J* = 7.4 Hz, CH_2_), 2.37 (3H, s, CH_3_); EIMS *m*/*z 364* [M]^+^ (7), 362 [M]^+^ (7), 238 (12), 237 (99), 236 (17), 235 (100), 225 (10), 224 (82), 223 (14), 222 (85), 209 (7), 156 (6), 144 (7), 143 (36), 142 (10), 128 (5), 115 (10), 111 (34). Anal. Calcd for C_16_H_15_BrN_2_OS: C 52.90; H 4.16; N 7.71%. Found: C 52.86; H 4.14; N 7.67%.

*N-[2-(2,5-Dimethyl-1H-indol-3-yl)ethyl]-2,3-dihydrothieno[3,4-b][1,4]dioxine-5-carboxamide (****23****)*

^1^H NMR (DMSO-*d*_6_) *δ* 10.31 (1H, s, NH), 7.17 (1H, s, H-4), 7.10 (1H, d, *J* = 8.1 Hz,H-7), 6.83 (1H, t, *J* = 5.8 Hz, NHCO), 6.77 (1H, d, *J* = 8.1 Hz, H-6), 6.57 (1H, s, H-7'), 4.19-4.09 (4H, m, H-2',3'), 3.50 (2H, q, *J* = 6.6 Hz, CH_2_N), 2.88 (2H, m,CH_2_), 2.36 (3H, s, CH_3_), 2.35 (3H, s, CH_3_); EIMS *m*/*z 356* [M]^+^ (14), 172 (8), 171 (86), 169 (26), 159 (9), 158 (100), 156 (17), 143 (13), 142 (6), 115 (10), 85 (16), 83 (18). Anal. Calcd for C_19_H_20_N_2_O_3_S: C 64.02; H 5.66; N 7.86%. Found: C 64.10; H 5.69; N 7.80%.

*N-[2-(2,5-Dimethyl-1H-indol-3-yl)ethyl]-5-ethylthieno[2,3-b]thiophene-2-carboxamide (****24****)*

^1^H NMR (DMSO-*d*_6_) *δ* 10.50 (1H, s, NH), 8.57 (1H, s, NHCO), 7.80 (1H, s, H-3'), 7.24 (1H, s, H-4), 7.11 (1H, s, H-4'), 7.10 (1H, d, *J* = 8.1 Hz,H-7), 6.79 (1H, d, *J* = 8.1 Hz,H-6), 3.38 (2H, q, *J* = 6.9 Hz, CH_2_N), 2.89 (2H, t, *J* = 7.4 Hz, CH_2_), 2.87 (2H, q, *J* = 7.5 Hz, CH_2_CH_3_-5'), 2.33 (3H, s, CH_3_), 2.29 (3H, s, CH_3_), 1.29 (3H, t, *J* = 7.5 Hz, CH_3_CH_2_-5'); EIMS *m*/*z 383* [M+1]^+^ (6), 382 [M]^+^ (25), 195 (12), 172 (13), 171 (95), 159 (13), 158 (100), 157 (11), 156 (7), 143 (9), 115 (5). Anal. Calcd for C_21_H_22_N_2_OS_2_: C 65.93; H 5.80; N 7.32%. Found: C 66.07; H 5.84; N 7.18%.

*N-[2-(2,5-Dimethyl-1H-indol-3-yl)ethyl]-3-nitrobenzamide (****25****)*

^1^H NMR (DMSO-*d*_6_) *δ* 10.19 (1H, s, NH), 8.72 (1H, d, *J* = 2.1 Hz,H-2'), 8.65 (1H, s, NHCO), 8.33-8.25 (2H, m, H-4',6'), 7.68 (1H, t, *J* = 8.0 Hz,H-5'), 7.21 (1H, s, H-4), 7.08 (1H, dd, *J* = 8.3 Hz, *J* = 2.1 Hz,H-7), 6.75 (1H, dd, *J* = 8.3 Hz, *J* = 1.7 Hz, H-6), 3.48 (2H, q, *J* = 6.7 Hz, CH_2_N), 2.93 (2H, t, *J* = 7.1 Hz, CH_2_), 2.37 (3H, s, CH_3_), 2.34 (3H, s, CH_3_); EIMS *m*/*z 337* [M]^+^ (9), 171 (35), 159 (12), 158 (100), 157 (23), 156 (18), 150 (23), 143 (9), 115 (12), 104 (14), 91 (5), 77 (5), 77 (5), 76 (19). Anal. Calcd for C_19_H_19_N_3_O_3_: C 67.64; H 5.68; N 12.45%. Found: C 67.75; H 5.71; N 12.34%.

*N-[2-(2,5-Dimethyl-1H-indol-3-yl)ethyl]-4-methylbenzamide (****26****)*

^1^H NMR (DMSO-*d*_6_) *δ* 10.18 (1H, s, NH), 7.99 (1H, s, NHCO), 7.71 (2H, d, *J* = 7.9 Hz, H-2',6'), 7.21 (1H, s, H-4), 7.17 (2H, d, *J* = 7.9 Hz, H-3',5'), 7.07 (1H, d, *J* = 8.1 Hz,H-7), 6.74 (1H, d, *J* = 8.1 Hz, H-6), 3.42 (2H, q, *J* = 6.6 Hz, CH_2_N), 2.89 (2H, t, *J* = 7.1 Hz, CH_2_), 2.39 (3H, s, CH_3_), 2.38 (3H, s, CH_3_), 2.34 (3H, s, CH_3_); EIMS *m*/*z 306* [M]^+^ (11), 172 (13), 171 (100), 159 (11), 158 (90), 157 (11), 156 (10), 143 (8), 119 (17), 115 (8), 91 (23). Anal. Calcd for C_20_H_22_N_2_O: C 78.40; H 7.24; N 9.14%. Found: C 78.55; H 7.27; N 9.00%.

*2-Chloro-N-[2-(2,5-dimethyl-1H-indol-3-yl)ethyl]-4,5-difluorobenzamide (****27****)*

^1^H NMR (DMSO-*d*_6_) *δ* 10.23 (1H, s, NH), 8.11 (1H, s, NHCO), 7.37 (1H, dd, *J* = 10.1 Hz, *J*_H-F_= 6.9 Hz,H-6'), 7.25 (1H, t, *J*_H-F_= 8.8 Hz,H-3'), 7.21 (1H, s, H-4), 7.08 (1H, d, *J* = 8.1 Hz, H-7), 6.76 (1H, d, *J* = 8.1 Hz, H-6), 3.43 (2H, q, *J* = 6.9 Hz, CH_2_N), 2.89 (2H, t, *J* = 7.3 Hz, CH_2_), 2.40 (3H, s, CH_3_), 2.37 (3H, s, CH_3_); EIMS *m*/*z 364* [M]^+^ (2), *362* [M]^+^ (6), 175 (5), 172 (6), 171 (43), 159 (12), 158 (100), 157 (10), 156 (8), 143 (7), 115 (8). Anal. Calcd for C_19_H_17_ClF_2_N_2_O: C 62.90; H 4.72; N 7.72%. Found: C 62.98; H 4.76; N 7.68%.

*2,4-Dichloro-5-[(dimethylamino)sulfonyl]-N-[2-(2,5-dimethyl-1H-indol-3-yl)ethyl]benzamide (****28****)*

^1^H NMR (DMSO-*d*_6_) *δ* 10.23 (1H, s, NH), 8.38 (1H, s, NHCO), 7.92 (1H, s, H-6'), 7.66 (1H, s, H-3'), 7.21 (1H, s, H-4), 7.08 (1H, d, *J* = 8.1 Hz,H-7), 6.75 (1H, dd, *J* = 8.1 Hz, *J* = 1.6 Hz,H-6), 3.46 (2H, q, *J* = 6.8 Hz, CH_2_N), 2.91 (2H, t, *J* = 7.3 Hz, CH_2_), 2.86 (6H, s, 2CH_3_), 2.40 (3H, s, CH_3_), 2.38 (3H, s, CH_3_); EIMS *m*/*z 469* [M]^+^ (2), *467* [M]^+^ (4), 172 (7), 171 (37), 159 (14), 158 (100), 157 (10), 156 (6), 143 (7). Anal. Calcd for C_21_H_23_Cl_2_N_3_O_3_S: C 53.85; H 4.95; N 8.97%. Found: C 53.79; H 4.92; N 8.93%.

*N-[2-(2,5-Dimethyl-1H-indol-3-yl)ethyl]-2-methyl-1,3-benzoxazole-7-carboxamide (****29****)*

^1^H NMR (DMSO-*d*_6_) *δ* 10.40 (1H, s, NH), 7.78 (1H, d, *J* = 7.7 Hz, *H*_arom_), 7.72 (2H, m,NHCO, *H*_arom_), 7.37 (1H, t, *J* = 7.8 Hz,H-5'), 7.20 (1H, s, H-4), 7.11 (1H, d, *J* = 8.1 Hz,H-7), 6.76 (1H, d, *J* = 8.1 Hz, H-6), 3.60 (2H, m, CH_2_N), 2.94 (2H, t, *J* = 7.2 Hz, CH_2_), 2.54 (3H, s, CH_3_-2'), 2.36 (3H, s, CH_3_), 2.30 (3H, s, CH_3_); EIMS *m*/*z 347* [M]^+^ (12), 172 (7), 171 (63), 160 (16), 159 (11), 158 (100), 156 (15), 143 (9), 132 (14), 115 (8), 91 (8). Anal. Calcd for C_21_H_21_N_3_O_2_: C 72.60; H 6.09; N 12.10%. Found: C 72.73; H 6.12; N 12.01%.

*5-Benzoyl-N-[2-(2,5-dimethyl-1H-indol-3-yl)ethyl]-2-furamide (****30****)*

^1^H NMR (DMSO-*d*_6_) *δ* 10.23 (1H, s, NH), 8.30 (1H, s, NHCO), 7.97(2H, d, *J* = 7.7 Hz, H-2'',6''), 7.65 (1H, t, *J* = 7.4 Hz, H-4''), 7.54(2H, t, *J* = 7.6 Hz, H-3'',5''), 7.30 (1H, d, *J* = 3.7 Hz, H-3'), 7.22 (1H, s, H-4), 7.20 (1H, d, *J* = 3.7 Hz, H-4'), 7.09 (1H, d, *J* = 8.1 Hz,H-7), 6.75 (1H, d, *J* = 8.1 Hz, H-6), 3.42 (2H, q, *J* = 6.3 Hz, CH_2_N), 2.90 (2H, t, *J* = 7.2 Hz, CH_2_), 2.38 (3H, s, CH_3_), 2.36 (3H, s, CH_3_); EIMS *m*/*z 386* [M]^+^ (10), 199 (7), 171 (36), 159 (8), 158 (100), 156 (13), 143 (7), 115 (9), 105 (7), 77 (16). Anal. Calcd for C_24_H_22_N_2_O_3_: C 74.59; H 5.74; N 7.25%. Found: C 74.69; H 5.77; N 7.18%.

*4-Amino-N-[2-(2,5-dimethyl-1H-indol-3-yl)ethyl]-1,2,5-oxadiazole-3-carboxamide 2-oxide (****31****)*

^1^H NMR (DMSO-*d*_6_) *δ* 10.48 (1H, s, NH), 8.28 (1H, s, NHCO), 7.24 (1H, s, H-4), 7.10 (1H, d, *J* = 8.0 Hz, H-7), 6.79 (1H, dd, *J* = 8.0 Hz, *J* = 1.7 Hz, H-6), 6.50 (2H, s, NH_2_), 3.46 (2H, q, *J* = 6.8 Hz, CH_2_N), 2.86 (2H, t, *J* = 7.4 Hz, CH_2_), 2.34 (3H, s, CH_3_), 2.31 (3H, s, CH_3_); EIMS *m*/*z 316* [M+1]^+^ (5), *315* [M]^+^ (33), 285 (2), 172 (19), 171 (10), 159 (12), 158 (100), 156 (8). Anal. Calcd for C_15_H_17_N_5_O_3_: C 57.14; H 5.43; N 22.21%. Found: C 57.07; H 5.40; N 22.16%.

*4-Fluoro-N-[2-(5-methoxy-2-methyl-1H-indol-3-yl)ethyl]benzamide (****32****)*

^1^H NMR (DMSO-*d*_6_) *δ* 10.35 (1H, s, NH), 8.40 (1H, s, NHCO), 7.90 (2H, dd, *J*_H-H_= 8.5 Hz, *J*_H-F_= 5.6 Hz,H-2',6'), 7.21 (2H, t, *J*_H-H_= 8.8 Hz, *J*_H-F_= 8.8 Hz,H-3', 5'), 7.10 (1H, d, *J* = 8.6 Hz,H-7), 6.97 (1H, d, *J* = 2.4 Hz, H-4), 6.61 (1H, dd, *J* = 8.5 Hz, *J* = 2.4 Hz, H-6), 3.73 (3H, s, OCH_3_), 3.42 (2H, q, *J* = 6.8 Hz, CH_2_N), 2.87 (2H, t, *J* = 7.5 Hz, CH_2_), 2.32 (3H, s, CH_3_); EIMS *m*/*z 327* [M+1]^+^ (7), 326 [M]^+^ (35), 188 (11), 187 (74), 175 (13), 174 (100), 159 (10), 144 (5), 131 (9), 130 (5), 123 (18), 95 (9). Anal. Calcd for C_19_H_19_FN_2_O_2_: C 69.92; H 5.87; N 8.58%. Found: C 70.03; H 5.90; N 8.47%.

*3,4-Dimethoxy-N-[2-(5-methoxy-2-methyl-1H-indol-3-yl)ethyl]benzamide (****33****)*

^1^H NMR (DMSO-*d*_6_) *δ* 10.37 (1H, s, NH), 8.25 (1H, s, NHCO), 7.45 (1H, d, *J* = 8.4 Hz, *H-*6'), 7.42 (1H, s, H-2'), 7.10 (1H, d, *J* = 8.6 Hz,H-7), 7.00-6.94 (2H, m, H-4,5'), 6.60 (1H, dd, *J* = 8.6 Hz, *J* = 2.4 Hz,H-6), 3.83 (3H, s, OCH_3_), 3.81 (3H, s, OCH_3_), 3.73 (3H, s, OCH_3_-5), 3.41 (2H, q, *J* = 7.0 Hz, CH_2_N), 2.87 (2H, t, *J* = 7.5 Hz, CH_2_), 2.32 (3H, s, CH_3_); EIMS *m*/*z 369* [M+1]^+^ (4), *368* [M]^+^ (21), 188 (14), 187 (100), 175 (8), 174 (64), 165 (13), 159 (10), 131 (8). Anal. Calcd for C_21_H_24_N_2_O_4_: C 68.46; H 6.57; N 7.60%. Found: C 68.51; H 6.60; N 7.53%.

*3,4,5-Trimethoxy-N-[2-(5-methoxy-2-methyl-1H-indol-3-yl)ethyl]benzamide (****34****)*

^1^H NMR (DMSO-*d*_6_) *δ* 10.38 (1H, s, NH), 8.33 (1H, s, NHCO), 7.15 (2H, s, H-2',6'), 7.10 (1H, d, *J* = 8.6 Hz,H-7), 6.96 (1H, d, *J* = 2.5 Hz,H-4), 6.60 (1H, dd, *J* = 8.6 Hz, *J* = 2.5 Hz,H-6), 3.83 (6H, s, 2OCH_3_-3',5'), 3.74 (3H, s, OCH_3_), 3.72 (3H, s, OCH_3_), 3.41 (2H, q, *J* = 6.8 Hz, CH_2_N), 2.87 (2H, t, *J* = 7.4 Hz, CH_2_), 2.33 (3H, s, CH_3_); EIMS *m*/*z 399* [M+1]^+^ (3), *398* [M]^+^ (19), 195 (10), 188 (13), 187 (100), 175 (9), 174 (64), 159 (9), 131 (6). Anal. Calcd for C_22_H_26_N_2_O_5_: C 66.32; H 6.58; N 7.03%. Found: C 66.41; H 6.60; N 6.88%.

*Methyl 4-{[2-(5-methoxy-2-methyl-1H-indol-3-yl)ethyl]carbamoyl}benzoate (****35****)*

^1^H NMR (DMSO-*d*_6_) *δ* 10.38 (1H, s, NH), 8.59 (1H, s, NHCO), 8.02 (2H, d, *J* = 8.1 Hz, *H-*2',6'), 7.94 (2H, d, *J* = 8.1 Hz, *H-*3',5'), 7.10 (1H, d, *J* = 8.6 Hz,H-7), 6.97 (1H, d, *J* = 2.3 Hz,H-4), 6.60 (1H, dd, *J* = 8.6 Hz, *J* = 2.3 Hz,H-6), 3.90 (3H, s, OCH_3_*-*4'), 3.73 (3H, s, OCH_3_-5), 3.44 (2H, q, *J* = 6.9 Hz, CH_2_N), 2.89 (2H, t, *J* = 7.5 Hz, CH_2_), 2.32 (3H, s, CH_3_); EIMS *m*/*z 367* [M+1]^+^ (4), *366* [M]^+^ (21), 188 (10), 187 (78), 175 (12), 174 (100), 163 (9), 159 (10), 131 (9). Anal. Calcd for C_21_H_22_N_2_O_4_: C 68.84; H 6.05; N 7.65%. Found: C 68.90; H 6.07; N 7.55%.

*2-Chloro-N-[2-(5-methoxy-2-methyl-1H-indol-3-yl)ethyl]-5-nitrobenzamide (****36****)*

^1^H NMR (DMSO-*d*_6_) *δ* 10.20 (1H, s, NH), 8.38 (1H, s, NHCO), 8.20 (1H, dd, *J* = 8.8 Hz, *J* = 2.8 Hz,H-4'), 8.13 (1H, d, *J* = 2.8 Hz,H-6'), 7.68 (1H, d, *J* = 8.8 Hz,H-3'), 7.09 (1H, dd, *J* = 8.8 Hz, *J* = 2.0 Hz,H-7), 6.95 (1H, d, *J* = 2.6 Hz, H-4), 6.58 (1H, dd, *J* = 8.8 Hz, *J* = 2.6 Hz, H-6 ), 3.78 (3H, d, *J* = 1.9 Hz, OCH_3_), 3.48 (2H, q, *J* = 6.8 Hz, CH_2_N), 2.93 (2H, t, *J* = 7.3 Hz, CH_2_), 2.39 (3H, d, *J* = 1.9 Hz, CH_3_-2); EIMS *m*/*z 389* [M]^+^ (2), *387* [M]^+^ (6), 187 (24), 184 (10), 175 (10), 174 (100), 172 (6), 159 (17), 138 (11), 131 (20), 130 (14). Anal. Calcd for C_19_H_18_ClN_3_O_4_: C 58.84; H 4.68; N 10.83%. Found: C 58.78; H 4.65; N 10.78%.

*N-[2-(5-Methoxy-2-methyl-1H-indol-3-yl)ethyl]-4-methyl-3-nitrobenzamide (****37****)*

^1^H NMR (DMSO-*d*_6_) *δ* 10.18 (1H, s, NH), 8.56 (1H, s, NHCO), 8.46 (1H, d, *J* = 2.0 Hz,H-2'), 8.07 (1H, dt, *J* = 8.1 Hz, *J* = 1.8 Hz,H-6'), 7.47 (1H, d, *J* = 7.8 Hz,H-5'), 7.08 (1H, dd, *J* = 8.8 Hz, *J* = 1.9 Hz,H-7), 6.93 (1H, d, *J* = 2.6 Hz, H-4), 6.57 (1H, dd, *J* = 8.5 Hz, *J* = 2.6 Hz, H-6 ), 3.75 (3H, t, *J* = 1.5 Hz, OCH_3_), 3.41 (2H, q, *J* = 6.7 Hz, CH_2_N), 2.90 (2H, t, *J* = 7.3 Hz, CH_2_), 2.62 (3H, s, CH_3_-4'), 2.34 (3H, d, *J* = 2.0 Hz, CH_3_-2); EIMS *m*/*z 367* [M]^+^ (10), 187 (40), 175 (10), 174 (100), 172 (10), 164 (13), 159 (18), 131 (20), 130 (15), 118 (12), 90 (15), 89 (20). Anal. Calcd for C_20_H_21_N_3_O_4_: C 65.38; H 5.76; N 11.14%. Found: C 65.24; H 5.70; N 11.23%.

*2,4-Dichloro-5-[(dimethylamino)sulfonyl]-N-[2-(5-methoxy-2-methyl-1H-indol-3-yl)ethyl]benzamide (****38****)*

^1^H NMR (DMSO-*d*_6_) *δ* 10.19 (1H, s, NH), 8.38 (1H, s, NHCO), 7.92 (1H, s, H-6'), 7.65 (1H, s, H-3'), 7.08 (1H, d, *J* = 8.5 Hz,H-7), 6.94 (1H, d, *J* = 2.4 Hz,H-4), 6.58 (1H, dd, *J* = 8.5 Hz, *J* = 2.4 Hz,H-6), 3.79 (3H, s, OCH_3_-5), 3.48 (2H, q, *J* = 6.8 Hz, CH_2_N), 2.92 (2H, t, *J* = 7.3 Hz, CH_2_), 2.87 (6H, s, 2CH_3_), 2.39 (3H, s, CH_3_); EIMS *m*/*z 485* [M]^+^ (1), *483* [M]^+^ (2), 187 (32), 175 (12(12), 174 (100), 159 (12), 131 (10). Anal. Calcd for C_21_H_23_Cl_2_N_3_O_4_S: C 52.07; H 4.79; N 8.67%. Found: C 51.95; H 4.75; N 8.61%.

*N-[2-(5-Methoxy-2-methyl-1H-indol-3-yl)ethyl]-3-(4-methoxyphenyl)isoxazole-5-carboxamide (****39****)*

^1^H NMR (DMSO-*d*_6_) *δ* 10.57 (1H, s, NH), 9.06 (1H, t, *J* = 5.9 Hz, NHCO), 7.86 (2H, d, *J* = 8.8 Hz,H-2'',6''), 7.53 (1H, s, H-4'), 7.11 (1H,d, *J* = 8.7 Hz, *H-7),* 7.09 (2H, d, *J* = 8.8 Hz,H-3'',5''), 6.99 (1H, d, *J* = 2.4 Hz, H-4), 6.61 (1H, dd, *J* = 8.7 Hz, *J* = 2.4 Hz, H-6 ), 3.83 (3H, s, OCH_3_), 3.72 (3H, s, OCH_3_), 3.41 (2H, t, *J* = 7.0 Hz, CH_2_N), 2.87 (2H, t, *J* = 7.4 Hz, CH_2_), 2.30 (3H, s, CH_3_); EIMS *m*/*z 405* [M]^+^ (2), 187 (20), 174 (100), 159 (11), 146 (11), 131 (8). Anal. Calcd for C_23_H_23_N_3_O_4_: C 68.13; H 5.72; N 10.36%. Found: C 68.24; H 5.76; N 10.23%.

*2-Fluoro-N-{2-[2-methyl-5-(trifluoromethoxy)-1H-indol-3-yl]ethyl}benzamide (****40****)*

^1^H NMR (DMSO-*d*_6_) *δ* 10.96 (1H, s, NH), 8.23 (1H, s, NHCO), 7.60 (1H, t, *J*_H-H_= 7.4 Hz, *J*_H-F_= 7.4 Hz,H-6'), 7.54-7.48 (1H, m, H-5'), 7.42 (1H, d, *J* = 2.3 Hz,H-4), 7.33-7.21 (3H, m, H-7,3',4'), 6.93 (1H, dd, *J* = 8.5 Hz, *J* = 2.3 Hz,H-6), 3.42 (2H, q, *J* = 6.9 Hz, CH_2_N), 2.90 (2H, t, *J* = 7.4 Hz, CH_2_), 2.36 (3H, s, CH_3_); EIMS *m*/*z 380* [M]^+^ (7), 242 (13), 241 (100), 240 (8), 229 (11), 228 (87), 227 (8), 158 (5), 131 (11), 130 (6), 123 (31), 95 (11). Anal. Calcd for C_19_H_16_F_4_N_2_O_2_: C 60.00; H 4.24; N 7.37%. Found: C 60.12; H 4.28; N 7.25%.

*2-Chloro-N-{2-[2-methyl-5-(trifluoromethoxy)-1H-indol-3-yl]ethyl}benzamide (****41****)*

^1^H NMR (DMSO-*d*_6_) *δ* 10.98 (1H, s, NH), 8.39 (1H, s, NHCO), 7.47 (1H, d, *J* = 8.0 Hz,H-6'), 7.44 – 7.39 (1H, m, H_arom_), 7.41 (1H, s, H-4), 7.34 (1H, t, *J* = 7.3 Hz,H_arom_), 7.32-7.28 (2H, m, H-7, H_arom_), 6.94 (1H, d, *J* = 8.4 Hz,H-6), 3.40 (2H, q, *J* = 6.9 Hz, CH_2_N), 2.89 (2H, t, *J* = 7.4 Hz, CH_2_), 2.38 (3H, s, CH_3_); EIMS *m*/*z* 398 [M]^+^ (2), 396 [M]^+^ (6), 242 (12), 241 (100), 229 (8), 228 (66), 227 (7), 139 (15), 131 (7), 111 (7). Anal. Calcd for C_19_H_16_ClF_3_N_2_O_2_: C 57.51; H 4.06; N 7.06%. Found: C 57.59 H 4.09; N 6.98%.

*3,4-Difluoro-N-{2-[2-methyl-5-(trifluoromethoxy)-1H-indol-3-yl]ethyl}benzamide (****42****)*

^1^H NMR (DMSO-*d*_6_) *δ* 10.90 (1H, s, NH), 8.56 (1H, s, NHCO), 7.81 (1H, ddd, *J* = 11.7 Hz, *J*_H-H_= 7.8 Hz, *J*_H-F_= 7.8 Hz, *J* = 2.2 Hz, H-2'), 7.70 (1H, ddd, *J* = 10.0 Hz, Hz, *J*_H-H_= 4.6 Hz, *J*_H-F_= 4.6 Hz, *J* = 2.1 Hz, H-6'), 7.45 (1H, dt, *J* = 10.5 Hz, *J*_H-H_= 8.3 Hz, *J*_H-F_= 8.3 Hz,H-5'), 7.35 (1H, d, *J* = 2.2 Hz, H-4), 7.27 (1H, d, *J* = 8.6 Hz,H-7), 6.89 (1H, dd, *J* = 8.6 Hz, *J* = 2.2 Hz, H-6), 3.41 (2H, q, *J* = 6.8 Hz, CH_2_N), 2.89 (2H, t, *J* = 7.3 Hz, CH_2_), 2.34 (3H, s, CH_3_); EIMS *m*/*z 398* [M]^+^ (7), 242 (12), 241 (87), 240 (11), 229 (13), 228 (100), 227 (10), 158 (6), 143 (5), 141 (26), 131 (13), 130 (6), 113 (18), 63 (9). Anal. Calcd for C_19_H_15_F_5_N_2_O_2_: C 57.29; H 3.80; N 7.03%. Found: C 57.36; H 3.84; N 6.93%.

*2-Chloro-4,5-difluoro-N-{2-[2-methyl-5-(trifluoromethoxy)-1H-indol-3-yl]ethyl}benzamide (****43****)*

^1^H NMR (DMSO-*d*_6_) *δ* 10.93 (1H, s, NH), 8.42 (1H, s, NHCO), 7.64 (1H, dd, *J* = 10.4 Hz, *J* = 7.0 Hz,H-6'), 7.37 (1H, d, *J* = 2.3 Hz, H-4), 7.35-7.26 (2H, m, H-7, 3'), 6.90 (1H, d, *J* = 8.5 Hz, H-6), 3.41 (2H, q, *J* = 6.9 Hz, CH_2_N), 2.89 (2H, t, *J* = 7.5 Hz, CH_2_), 2.38 (3H, s, CH_3_); EIMS *m*/*z 434* [M]^+^ (2), 4*32* [M]^+^ (6), 242 (11), 241 (86), 240 (9), 229 (12), 228 (100), 227 (11), 175 (13), 158 (5), 147 (6), 131 (11), 130 (5). Anal. Calcd for C_19_H_14_ClF_5_N_2_O_2_: C 52.73; H 3.26; N 6.47%. Found: C 52.68; H 3.24; N 6.42%.

*N-{2-[2-Methyl-5-(trifluoromethoxy)-1H-indol-3-yl]ethyl}-3-nitrobenzamide (****44****)*

^1^H NMR (DMSO-*d*_6_) *δ* 10.70 (1H, s, NH), 8.73 (2H, m,NHCO, H-2'), 8.32-8.26 (2H, m, H-4',6'), 7.68 (1H, t, *J* = 7.9 Hz,H-5'), 7.34 (1H, s, H-4), 7.25 (1H, d, *J* = 8.7 Hz,H-7), 6.85 (1H, dd, *J* = 8.7 Hz, *J* = 2.3 Hz, H-6), 3.49 (2H, q, *J* = 6.5 Hz, CH_2_N), 2.95 (2H, t, *J* = 7.3 Hz, CH_2_), 2.38 (3H, s, CH_3_); EIMS *m*/*z 407* [M]^+^ (7), 242 (6), 241 (67), 229 (9), 228 (100), 158 (9), 150 (18), 131 (14), 130 (11), 104 (15), 103 (5), 77 (5), 76 (21). Anal. Calcd for C_19_H_16_F_3_N_3_O_4_: C 56.02; H 3.96; N 10.32%. Found: C 56.08; H 3.98; N 10.28%.

*2-Chloro-N-{2-[2-methyl-5-(trifluoromethoxy)-1H-indol-3-yl]ethyl}-5-nitrobenzamide (****45****)*

^1^H NMR (DMSO-*d*_6_) *δ* 11.00 (1H, s, NH), 8.67 (1H, t, *J* = 5.8 Hz,NHCO), 8.25 (1H, m, H-4'), 8.06 (1H, d, *J* = 2.4 Hz,H-6'), 7.79 (1H, dd, *J* = 8.7 Hz, *J* = 1.9 Hz,H-3'), 7.40 (1H, s, H-4), 7.31 (1H, dd, *J* = 8.7 Hz, *J* = 2.0 Hz, H-7), 6.93 (1H, d, *J* = 8.7 Hz, H-6), 3.45 (2H, q, *J* = 7.0 Hz, CH_2_N), 2.92 (2H, m, CH_2_), 2.38 (3H, d, *J* = 2.2 Hz, CH_3_); EIMS *m*/*z 443* [M]^+^ (1), 441 [M]^+^ (3), 242 (9), 241 (64), 240 (7), 229 (11), 228 (100), 184 (6), 138 (6), 131 (9). Anal. Calcd for C_19_H_15_ClF_3_N_3_O_4_: C 51.66; H 3.42; N 9.51%. Found: C 51.60; H 3.40; N 9.46%.

*2-Methoxy-N-{2-[2-methyl-5-(trifluoromethoxy)-1H-indol-3-yl]ethyl}benzamide (****46****)*

^1^H NMR (DMSO-*d*_6_) *δ* 11.02 (1H, s, NH), 8.14 (1H, t, *J* = 5.7 Hz,NHCO), 7.80 (1H, dd, *J* = 7.7 Hz, *J* = 1.9 Hz,H-6'), 7.48 – 7.41 (1H, m, H-4'), 7.44 (1H, s, H-4), 7.31 (1H, d, *J* = 8.6 Hz,H-7), 7.10 (1H, d, *J* = 8.3 Hz, *H-3*'), 7.02 (1H, t, *J* = 7.5 Hz, H-5'), 6.94 (1H, dd, *J* = 8.6 Hz, *J* = 2.3 Hz,H-6), 3.75 (3H, s, OCH_3_), 3.47 (2H, q, *J* = 6.8 Hz, CH_2_N), 2.89 (2H, t, *J* = 7.2 Hz, CH_2_), 2.36 (3H, s, CH_3_); EIMS *m*/*z* 392 [M]^+^ (5), 242 (12), 241 (100), 229 (5), 228 (50), 158 (5), 152 (25), 136 (5), 135 (64), 131 (9), 92 (9), 77 (23). Anal. Calcd for C_20_H_19_F_3_N_2_O_3_: C 61.22; H 4.88; N 7.14%. Found: C 61.27 H 4.90; N 7.10%.

*4-Methoxy-N-{2-[2-methyl-5-(trifluoromethoxy)-1H-indol-3-yl]ethyl}benzamide (****47****)*

^1^H NMR (DMSO-*d*_6_) *δ* 10.99 (1H, s, NH), 8.39 (1H, t, *J* = 5.8 Hz,NHCO), 7.80 (2H, d, *J* = 8.5 Hz, *H-*2',6'), 7.41 (1H, d, *J* = 2.2 Hz,H-4), 7.29 (1H, d, *J* = 8.6 Hz,H-7), 6.97 (2H, d, *J* = 8.5 Hz, *H-*3',5'), 6.93 (1H, d, *J* = 8.6 Hz,H-6), 3.81 (3H, s, OCH_3_), 3.38 (2H, q, *J* = 6.8 Hz, CH_2_N), 2.87 (2H, t, *J* = 7.4 Hz, CH_2_), 2.32 (3H, s, CH_3_); EIMS *m*/*z* *392* [M]^+^ (5), 242 (12), 241 (100), 229 (6), 228 (45), 227 (6), 152 (9), 135 (46), 131 (7), 107 (5), 92 (7), 77 (13). Anal. Calcd for C_20_H_19_ F_3_N_2_O_3_: C 61.22; H 4.88; N 7.14%. Found: C 61.29; H 4.91; N 7.02%.

*3,4-Dimethoxy-N-{2-[2-methyl-5-(trifluoromethoxy)-1H-indol-3-yl]ethyl}benzamide (****48****)*

^1^H NMR (DMSO-*d*_6_) *δ* 10.97 (1H, s, NH), 8.37 (1H, s, NHCO), 7.45 (2H, d, *J* = 8.2 Hz, *H-*6'), 7.42 (1H, d, *J* = 1.3 Hz,H-4), 7.41 (1H, s, H-2'), 7.30 (1H, d, *J* = 8.6 Hz,H-7), 7.00 (1H, d, *J* = 8.2 Hz, *H-*5'), 6.93 (1H, dd, *J* = 8.6 Hz, *J* = 1.3 Hz,H-6), 3.81 (3H, s, OCH_3_), 3.79 (3H, s, OCH_3_), 3.39 (2H, q, *J* = 6.8 Hz, CH_2_N), 2.88 (2H, t, *J* = 7.3 Hz, CH_2_), 2.33 (3H, s, CH_3_); EIMS *m*/*z* 42*2* [M]^+^ (7), 242 (13), 241 (100), 229 (5), 228 (38), 182 (7), 165 (35), 131 (6), 79 (7), 77 (9). Anal. Calcd for C_21_H_21_ F_3_N_2_O_4_: C 59.71; H 5.01; N 6.63%. Found: C 59.80; H 5.04; N 6.52%.

*3,4,5-Trimethoxy-N-{2-[2-methyl-5-(trifluoromethoxy)-1H-indol-3-yl]ethyl}benzamide (****49****)*

^1^H NMR (DMSO-*d*_6_) *δ* 10.87 (1H, s, NH), 8.35 (1H, s,NHCO), 7.37 (1H, s,H-4), 7.28 (1H, d, *J* = 8.6 Hz,H-7), 7.14 (2H, s, *H-2*',6'), 6.89 (1H, d, *J* = 8.6 Hz,H-6), 3.83 (6H, s, 2OCH_3_), 3.73 (3H, s, OCH_3_), 3.40 (2H, q, *J* = 6.9 Hz, CH_2_N), 2.90 (2H, t, *J* = 7.5 Hz, CH_2_), 2.36 (3H, s, CH_3_); EIMS *m*/*z* 452 [M]^+^ (6), 242 (13), 241 (100), 229 (6), 228 (41), 212 (6), 195 (28), 131 (7), 81 (5), 77 (6). Anal. Calcd for C_22_H_23_ F_3_N_2_O_5_: C 58.41; H 5.12; N 6.19%. Found: C 58.49; H 5.15; N 6.09%.

*Methyl 4-({2-[2-methyl-5-(trifluoromethoxy)-1H-indol-3-yl]ethyl}carbamoyl)benzoate (****50****)*

^1^H NMR (DMSO-*d*_6_) *δ* 10.96 (1H, s, NH), 8.68 (1H, s,NHCO), 8.01 (2H, d, *J* = 8.2 Hz, *H-*2',6'), 7.92 (2H, d, *J* = 8.2 Hz, *H-3*',5'), 7.40 (1H, s, H-4), 7.29 (1H, d, *J* = 8.7 Hz,H-7), 6.92 (1H, d, *J* = 8.7 Hz,H-6), 3.89 (3H, s, OCH_3_), 3.43 (2H, q, *J* = 6.7 Hz, CH_2_N), 2.91 (2H, t, *J* = 7.3 Hz, CH_2_), 2.33 (3H, s, CH_3_); EIMS *m*/*z* 420 [M]^+^ (4), 389 (1), 242 (14), 241 (100), 240 (10), 229 (10), 228 (79), 227 (9), 163 (13), 135 (5), 131 (9), 104 (7), 103 (5). Anal. Calcd for C_21_H_19_ F_3_N_2_O_4_: C 60.00; H 4.56; N 6.66%. Found: C 59.93; H 4.53; N 6.63%.

*2-Methyl-N-{2-[2-methyl-5-(trifluoromethoxy)-1H-indol-3-yl]ethyl}-1,3-benzoxazole-7-carboxamide (****51****)*

^1^H NMR (DMSO-*d*_6_) *δ* 10.85 (1H, s, NH), 7.87 (1H, s,NHCO), 7.74 (1H, d, *J* = 7.8 Hz,H_arom_), 7.70 (1H, d, *J* = 7.8 Hz,H_arom_ ), 7.37 (1H, s, H-4), 7.36 (1H, t, *J* = 7.8 Hz, *H-5*'*), 7.27 (1H, d, J* = 8.6 Hz, H-7), 6.86 (1H, d, *J* = 8.6 Hz, H-6), 3.56 (2H, q, *J* = 6.7 Hz, CH_2_N), 2.97 (2H, t, *J* = 7.3 Hz, CH_2_), 2.60 (3H, s, CH_3_*-2*')2.41 (3H, s, CH_3_*-*2); EIMS *m*/*z 417* [M]^+^ (14), 242 (8), 241 (100), 229 (9), 228 (99), 160 (46), 158 (9), 143 (5), 132 (25), 131 (19), 130 (11), 104 (6), 91 (10), 77 (7). Anal. Calcd for C_21_H_18_F_3_N_3_O_3_: C 60.43; H 4.53; N 10.07%. Found: C 60.49; H 4.57; N 10.00%.

*4-{[Bis(2-chloroethyl)amino]sulfonyl}-N-{2-[2-methyl-5-(trifluoromethoxy)-1H-indol-3-yl]ethyl}benzamide (****52****)*

^1^H NMR (DMSO-*d*_6_) *δ* 10.98 (1H, s, NH), 8.78 (1H, t, *J* = 5.8 Hz, NHCO), 8.00 (2H, d, *J* = 8.2 Hz, H-2',6'), 7.95 (2H, d, *J* = 8.2 Hz, H-3',5'), 7.39 (1H, s, H-4), 7.29 (1H, d, *J* = 8.5 Hz, H-7), 6.92 (1H, d, *J* = 8.7 Hz, H-6), 3.73 (4H, t, *J* = 6.7 Hz, 2CH_2_Cl), 3.52 (4H, t, *J* = 6.7 Hz, 2CH_2_N), 3.43 (2H, q, *J* = 6.9 Hz, CH_2_N), 2.90 (2H, t, *J* = 7.3 Hz, CH_2_), 2.33 (3H, s, CH_3_); EIMS *m*/*z* 242 (10), 241 (80), 229 (13), 228 (100), 175 (9), 162 (13), 131 (9), 105 (8), 104 (27), 76 (13). Anal. Calcd for C_23_H_24_Cl_2_F_3_N_3_O_4_S: C 48.77; H 4.27; N 7.42%. Found: C 48.73; H 4.22; N 7.38%.

*5-Bromo-N-{2-[2-methyl-5-(trifluoromethoxy)-1H-indol-3-yl]ethyl}-2-furamide (****53****)*

^1^H NMR (DMSO-*d*_6_) *δ* 10.97 (1H, s, NH), 8.43 (1H, t, *J* = 5.9 Hz,NHCO), 7.39 (1H, d, *J* = 2.3 Hz,H-4), 7.29 (1H, d, *J* = 8.6 Hz,H-7), 7.07 (1H, d, *J* = 3.5 Hz,H-*3*'), 6.92 (1H, dd, *J* = 8.6 Hz, *J* = 2.3 Hz,H-6), 6.72 (1H, d, *J* = 3.5 Hz,H-4'), 3.34 (2H, q, *J* = 6.8 Hz, CH_2_N), 2.85 (2H, t, *J* = 7.4 Hz, CH_2_), 2.33 (3H, s, CH_3_); EIMS *m*/*z* 432 [M]^+^ (7), 430 [M]^+^ (7), 242 (14), 241 (98), 240 (11), 229 (12), 228 (100), 227 (11), 175 (9), 173 (10), 158 (5), 131 (10), 130 (5). Anal. Calcd for C_17_H_14_BrF_3_N_2_O_3_: C 47.35; H 3.27; N 6.50%. Found: C 47.30 H 3.23; N 6.45%.

*4-Chloro-1-methyl-N-{2-[2-methyl-5-(trifluoromethoxy)-1H-indol-3-yl]ethyl}-1H-pyrazole-5-carboxamide (****54****)*

^1^H NMR (DMSO-*d*_6_) *δ* 10.99 (1H, s, NH), 8.30 (1H, s,NHCO), 7.56 (1H, s, *H-3*'),7.42 (1H, d, *J* = 2.3 Hz,H-4), 7.30 (1H, d, *J* = 8.6 Hz,H-7), 6.93 (1H, dd, *J* = 8.6 Hz, *J* = 2.3 Hz,H-6), 3.81 (3H, s, NCH_3_), 3.45 (2H, q, *J* = 6.9 Hz, CH_2_N), 2.90 (2H, t, *J* = 7.4 Hz, CH_2_), 2.36 (3H, s, CH_3_); EIMS *m*/*z* 402 [M]^+^ (2), 400 [M]^+^ (6), 242 (8), 241 (55), 240 (6), 229 (12), 228 (100), 227 (11), 143 (16), 131 (10), 130 (5). Anal. Calcd for C_17_H_16_ClF_3_N_4_O_2_: C 50.95; H 4.02; N 13.98%. Found: C 50.87; H 4.00; N 14.12%.

*4-Amino-N-{2-[2-methyl-5-(trifluoromethoxy)-1H-indol-3-yl]ethyl}-1,2,5-oxadiazole-3-carboxamide 2-oxide (****55****)*

^1^H NMR (DMSO-*d*_6_) *δ* 10.96 (1H, s, NH), 8.34 (1H, t, *J* = 5.7 Hz, NHCO), 7.42 (1H, d, *J* = 2.3 Hz, H-4), 7.29 (1H, d, *J* = 8.7 Hz, H-7), 6.92 (1H, d, *J* = 8.7 Hz, H-6), 6.49 (2H, s, NH_2_), 3.46 (2H, q, *J* = 7.0 Hz, CH_2_N), 2.89 (2H, t, *J* = 7.5 Hz, CH_2_), 2.35 (3H, s, CH_3_); EIMS *m*/*z 386* [M+1]^+^ (1), *315* [M]^+^ (33), 301 (5), 242 (32), 241 (25), 229 (11), 228 (100), 131 (7), 130 (5). Anal. Calcd for C_15_H_14_F_3_N_5_O_4_: C 46.76; H 3.66; N 18.18%. Found: C 46.70; H 3.62; N 18.12%.

*N-{2-[5-(Benzyloxy)-2-methyl-1H-indol-3-yl]ethyl}-2-methoxybenzamide (****56****)*

^1^H NMR (DMSO-*d*_6_) *δ* 10.47 (1H, s, NH), 8.00 (1H, s, NHCO), 7.90 (1H, dd, *J* = 7.8 Hz, *J* = 2.0 Hz, *H-*6''), 7.46-7.41 (3H, m, H*-*2',6', H_arom_), 7.36 (2H, t, *J* = 7.5 Hz, *H-*3',5'), 7.29 (1H, t, *J* = 7.3 Hz, *H-4*'), 7.14 (1H, d, *J* = 8.6 Hz,H-7), 7.08 (1H, d, *J* = 2.3 Hz, H-4), 7.07-6.99 (2H, m, H*-3*'', H_arom_), 6.70 (1H, dd, *J* = 8.6 Hz, *J* = 2.3 Hz,H-6), 6.67 (1H, dd, *J* = 8.6 Hz, *J* = 2.4 Hz,H-6), 5.00 (2H, s, OCH_2_), 3.69 (3H, s, OCH_3_), 3.40 (2H, q, *J* = 6.7 Hz, CH_2_N), 2.88 (2H, t, *J* = 7.1 Hz, CH_2_), 2.34 (3H, s, CH_3_); EIMS *m*/*z 415* [M+1]^+^ (7), 414 [M]^+^ (27), 264 (20), 263 (100), 251 (6), 250 (41), 173 (8), 172 (49), 160 (11), 159 (23), 144 (6), 135 (64), 131 (13), 130 (6), 92 (12), 91 (61), 77 (12). Anal. Calcd for C_26_H_26_N_2_O_3_: C 75.34; H 6.32; N 6.76%. Found: C 75.44; H 6.35; N 6.70%.

*N-{2-[5-(Benzyloxy)-2-methyl-1H-indol-3-yl]ethyl}-3,4-dimethoxybenzamide (****57****)*

^1^H NMR (DMSO-*d*_6_) *δ* 10.41 (1H, s, NH), 8.25 (1H, s, NHCO), 7.48-7.40 (4H, m, H*-*2',6',2'',6''), 7.37 (2H, t, *J* = 7.5 Hz, *H-*3',5'), 7.30 (1H, t, *J* = 7.3 Hz, *H-4*'), 7.11 (1H, d, *J* = 8.6 Hz,H-7), 7.07 (1H, s, H-4), 6.95 (1H, dd, *J* = 8.4 Hz, *J* = 2.5 Hz,H-5''), 6.67 (1H, dd, *J* = 8.6 Hz, *J* = 2.4 Hz,H-6), 4.97 (2H, s, OCH_2_), 3.81 (3H, s, OCH_3_), 3.79 (3H, s, OCH_3_), 3.40 (2H, q, *J* = 6.9 Hz, CH_2_N), 2.86 (2H, t, *J* = 7.4 Hz, CH_2_), 2.32 (3H, s, CH_3_); EIMS *m*/*z 445* [M+1]^+^ (5), 444 [M]^+^ (22), 264 (18), 263 (100), 251 (10), 250 (61), 172 (47), 171 (18), 165 (60), 160 (9), 159 (33), 144 (9), 131 (15), 130 (10), 91 (53), 77 (7), 76 (12). Anal. Calcd for C_27_H_28_N_2_O_4_: C 72.95; H 6.35; N 6.30%. Found: C 73.10; H 6.35; N 6.18%.

*Methyl 4-({2-[5-(benzyloxy)-2-methyl-1H-indol-3-yl]ethyl}carbamoyl)benzoate (****58****)*

^1^H NMR (DMSO-*d*_6_) *δ* 10.41 (1H, s, NH), 8.61 (1H, s, NHCO), 8.01 (2H, d, *J* = 8.2 Hz, *H-*2'',6''), 7.94 (2H, d, *J* = 8.2 Hz, *H-*3'',5''), 7.43 (2H, d, *J* = 7.6 Hz, *H-*2',6'), 7.37 (2H, t, *J* = 7.6 Hz, *H-*3',5'), 7.30 (1H, t, *J* = 7.3 Hz, *H-4*'),7.11 (1H, d, *J* = 8.6 Hz,H-7), 7.07 (1H, s, H-4), 6.68 (1H, dd, *J* = 8.6 Hz, *J* = 2.4 Hz,H-6), 4.98 (2H, s, OCH_2_), 3.89 (3H, s, OCH_3_), 3.43 (2H, q, *J* = 6.8 Hz, CH_2_N), 2.88 (2H, t, *J* = 7.4 Hz, CH_2_), 2.31 (3H, s, CH_3_); EIMS *m*/*z 443* [M+1]^+^ (5), 442 [M]^+^ (19), 264 (12), 263 (66), 251 (13), 250 (84), 188 (120), 173 (10), 172 (65), 171 (26), 163 (55), 160 (23), 159 (54), 144 (18), 135 (16), 131 (35), 130 (20), 104 (14), 103 (20), 91 (100), 77 (11), 76 (12). Anal. Calcd for C_27_H_26_N_2_O_4_: C 73.29; H 5.92; N 6.33%. Found: C 73.45; H 5.96; N 6.18%.

*N-{2-[5-(Benzyloxy)-2-methyl-1H-indol-3-yl]ethyl}-3-bromobenzamide (****59****)*

^1^H NMR (DMSO-*d*_6_) *δ* 10.41 (1H, s, NH), 8.56 (1H, s, NHCO), 8.00 (1H, d, *J* = 1.8 Hz, *H-2*''), 7.83 (1H, dd, *J* = 7.9 Hz, *J* = 1.4 Hz, *H-*6''), 7.65 (1H, dd, *J* = 8.3 Hz, *J* = 2.0 Hz, *H-4*''), 7.45 (2H, d, *J* = 7.6 Hz, H*-*2',6'), 7.41-7.34 (3H, m, *H-*3',5', *5*''), 7.30 (1H, t, *J* = 7.3 Hz, *H-4*'), 7.11 (1H, d, *J* = 8.6 Hz,H-7), 7.06 (1H, d, *J* = 2.0 Hz, H-4), 6.67 (1H, dd, *J* = 8.6 Hz, *J* = 2.4 Hz,H-6), 5.00 (2H, s, OCH_2_), 3.41 (2H, q, *J* = 6.9 Hz, CH_2_N), 2.86 (2H, t, *J* = 7.1 Hz, CH_2_), 2.31 (3H, s, CH_3_); EIMS *m*/*z 464* [M]^+^ (9), 462 [M]^+^ (9), 264 (13), 263 (68), 251 (18), 250 (100), 188 (12), 185 (15), 183 (14), 173 (9), 172 (49), 171 (22), 160 (15), 159 (38), 157 (8), 155 (7), 144 (10), 131 (20), 130 (10), 91 (29). Anal. Calcd for C_25_H_23_BrN_2_O_2_: C 64.80; H 5.00; N 6.05%. Found: C 64.88; H 5.05; N 6.00%.

*2,4-Dichloro-N-[2-(5-fluoro-2-methyl-1H-indol-3-yl)ethyl]benzamide (****60****)*

^1^H NMR (DMSO-*d*_6_) *δ* 10.76 (1H, s, NH), 8.44 (1H, s, NHCO), 7.63 (1H, d, *J* = 2.0 Hz,H-3'), 7.44 (1H, dd, *J* = 8.2 Hz, *J* = 2.0 Hz, H-5'), 7.34 (1H, d, *J* = 8.2 Hz, H-6'), 7.22-7.17 (2H, m, H-4,7), 6.79 (1H, td, *J*_H-H_= 9.2 Hz, *J*_H-F_= 9.2 Hz, *J* = 2.5 Hz, H-6), 3.38 (2H, q, *J* = 6.8 Hz, CH_2_N), 2.85 (2H, t, *J* = 7.5 Hz, CH_2_), 2.34 (3H, s, CH_3_); EIMS *m*/*z 366* [M]^+^ (1), *364* [M]^+^ (2), 176 (9), 175 (89, 173 (14), 163 (9), 162 (100), 161 (9), 147 (6), 133 (6), 109 (7). Anal. Calcd for C_18_H_15_ Cl_2_FN_2_O: C 59.19; H 4.14; N 7.67%. Found: C 59.27; H 4.18; N 7.57%.

*N-[2-(5-Fluoro-2-methyl-1H-indol-3-yl)ethyl]-3-nitrobenzamide (****61****)*

^1^H NMR (DMSO-*d*_6_) *δ* 10.48 (1H, s, NH), 8.73 (2H, s, *NHCO,* H-2'), 8.34-8.25 (2H, m, H-4',6'), 7.69 (1H, t, *J* = 7.9 Hz,H-5'), 7.14 (2H, ddd, *J* = 12.3 Hz, *J* = 9.3 Hz, *J* = 2.1 Hz,H-4,7), 6.69 (1H, td, *J*_H-H_= 9.1 Hz, *J*_H-F_= 9.1 Hz, *J* = 2.5 Hz, H-6), 3.47 (2H, q, *J* = 6.9 Hz, CH_2_N), 2.91 (2H, t, *J* = 7.4 Hz, CH_2_), 2.36 (3H, s, CH_3_); EIMS *m*/*z 341* [M]^+^ (5), 175 (44), 162 (100), 150 (7), 104 (6), 76 (6). Anal. Calcd for C_18_H_16_FN_3_O_3_: C 63.34; H 4.72; N 12.31%. Found: C 63.43; H 4.74; N 12.21%.

*N-[2-(5-Fluoro-2-methyl-1H-indol-3-yl)ethyl]-3,5-dinitrobenzamide (****62****)*

^1^H NMR (DMSO-*d*_6_) *δ* 10.51 (1H, s, NH), 9.09 (3H, d, *J* = 1.9 Hz, *H-2*'*,4*'*,6*'*),* 8.98 (1H, t, *J* = 1.2 Hz,NHCO), 7.15 (1H, ddd, *J* = 25.9 Hz, *J*_H-F_= 9.1 Hz, *J* = 3.4 Hz,H-4), 7.10 (1H, dd, *J* = 9.2 Hz, *J* = 2.8 Hz, H-7), 6.69 (1H, td, *J*_H-H_= 9.2 Hz, *J*_H-F_= 9.2 Hz, *J* = 2.8 Hz, H-6), 3.51(2H, q, *J* = 6.8 Hz, CH_2_N), 2.93 (2H, t, *J* = 7.3 Hz, CH_2_), 2.36 (3H, s, CH_3_); EIMS *m*/*z 386* [M]^+^ (4), 175 (23), 162 (100), 75 (8). Anal. Calcd for C_18_H_15_FN_4_O_5_: C 55.96; H 3.91; N 14.50%. Found: C 55.90; H 3.88; N 14.56%.

*N-[2-(5-Fluoro-2-methyl-1H-indol-3-yl)ethyl]-2-methyl-1,3-benzoxazole-7-carboxamide (****63****)*

^1^H NMR (DMSO-*d*_6_) *δ* 10.65 (1H, s, NH), 7.87 (1H, s, NHCO), 7.75 (1H, d, *J* = 7.8 Hz, *H*_arom_), 7.71 (1H, d, *J* = 7.8 Hz, *H*_arom_), 7.36 (1H, t, *J* = 7.8 Hz,H-5'), 7.22-7.15 (2H, m, H-4,7), 6.72 (1H, td, *J*_H-H_= 9.1 Hz, *J*_H-F_= 9.1 Hz, *J* = 2.4 Hz, H-6), 3.56 (2H, q, *J* = 6.8 Hz,CH_2_N), 2.94 (2H, t, *J* = 7.3 Hz, CH_2_), 2.61 (3H, s, CH_3_-2'), 2.38 (3H, s, CH_3_-2); EIMS *m*/*z 351* [M]^+^ (11), 176 (8), 175 (100), 163 (8), 162 (89), 161 (25), 160 (32), 146 (5), 133 (7), 132 (19), 91 (6). Anal. Calcd for C_20_H_18_FN_3_O_2_: C 68.36; H 5.16; N 11.96%. Found: C 68.47; H 5.20; N 11.84%.

*2,4-Dichloro-5-[(dimethylamino)sulfonyl]-N-[2-(5-fluoro-2-methyl-1H-indol-3-yl)ethyl]benzamid*e (**64**)

^1^H NMR (DMSO-*d*_6_) *δ* 10.77 (1H, s, NH), 8.64 (1H, s, NHCO), 7.95 (1H, s, H-6'), 7.84 (1H, d, *J* = 2.9 Hz,H-3'), 7.20 (2H, dt, *J* = 8.1 Hz, *J* = 3.5 Hz,H-4,7), 6.79 (1H, t, *J*_H-H_= 9.2 Hz, *J*_H-F_= 9.2 Hz, H-6), 3.43 (2H, q, *J* = 6.8 Hz, CH_2_N), 2.88 (2H, t, *J* = 7.9 Hz, CH_2_), 2.83 (6H, s, 2CH_3_), 2.36 (3H, s, CH_3_); EIMS *m*/*z 475* [M]^+^ (1), *473* [M]^+^ (4), *471* [M]^+^ (6), 176 (9), 175 (69), 174 (6), 163 (13), 162 (100), 161 (9). Anal. Calcd for C_20_H_20_Cl_2_FN_3_O_3_S: C 50.85; H 4.27; N 8.90%. Found: C 50.79; H 4.23; N 8.85%.

*4-{[Bis(2-chloroethyl)amino]sulfonyl}-N-[2-(5-fluoro-2-methyl-1H-indol-3-yl)ethyl]benzamide (****65****)*

^1^H NMR (DMSO-*d*_6_) *δ* 10.76 (1H, s, NH), 8.78 (1H, NHCO), 8.02 (2H, *J* = 8.4 Hz, H-2',6'), 7.96 (2H, *J* = 8.4 Hz, H-3',5'), 7.24-7.15 (2H, m, H-4,7), 6.79 (1H, td, *J*_H-H_= 9.2 Hz, *J*_H-F_= 9.2 Hz, *J* = 2.5 Hz, H-6), 3.73 (4H, t, *J* = 6.8 Hz, 2CH_2_Cl), 3.52 (4H, t, *J* = 6.8 Hz, 2CH_2_N), 3.42 (2H, q, *J* = 6.9 Hz, CH_2_N), 2.87 (2H, t, *J* = 7.3 Hz, CH_2_), 2.31 (3H, s, CH_3_); EIMS *m*/*z 501* [M]^+^ (1), 499[M]^+^ (2), 176 (9), 175 (72), 163 (10), 162 (100), 161 (14), 13 (5), 105 (5), 104 (21), 76 (12). Anal. Calcd for C_22_H_24_Cl_2_FN_3_O_3_S: C 52.80; H 4.83; N 8.40%. Found: C 52.87; H 4.87; N 8.25%.

*N-[2-(5-Fluoro-2-methyl-1H-indol-3-yl)ethyl]-2-(4-methylphenyl)-1,3-dioxoisoindoline-5-carboxamide (****66****)*

^1^H NMR (DMSO-*d*_6_) *δ* 10.79 (1H, s, NH), 8.96 (1H, t, *J* = 5.7 Hz,NHCO), 8.34 (1H, s, H-4'), 8.33 (1H, d, *J* = 8.6 Hz,H-6'), 8.04 (1H, d, *J* = 7.7 Hz,H-7'), 7.34 (4H, s, H-2'',3'',5'',6''), 7.24-7.18 (2H, m, H-4,7), 6.80 (1H, td, *J*_H-H_= 9.2 Hz, *J*_H-F_= 9.2 Hz, *J* = 2.5 Hz, H-6), 3.46(2H, q, *J* = 6.8 Hz, CH_2_N), 2.91 (2H, t, *J* = 7.3 Hz, CH_2_), 2.38 (3H, s, CH_3_), 2.32 (3H, s, CH_3_); EIMS *m*/*z 455* [M]^+^ (1), 176 (9), 175 (68), 174 (6), 173 (6), 163 (12), 162 (100), 161 (13), 160 (14), 150 (11), 146 (5), 103 (18), 75 (12). Anal. Calcd for C_27_H_22_FN_3_O_3_: C 71.20; H 4.87; N 9.23%. Found: C 71.32; H 4.91; N 9.10%.

*N-[2-(7-Chloro-2-methyl-1H-indol-3-yl)ethyl]-3,5-dinitrobenzamide (****67****)*

^1^H NMR (DMSO-*d*_6_) *δ* 10.70 (1H, s, NH), 9.08 (3H, d, *J* = 1.9 Hz, *H-2*'*,4*'*,6*'*),* 8.98 (1H, t, *J* = 2.1 Hz,NHCO), 7.39 (1H, d, *J* = 7.8 Hz,H-4), 6.96 (1H, d, *J* = 7.5 Hz,H-6), 6.88 (1H, t, *J* = 7.7 Hz, H-5), 3.52 (2H, q, *J* = 6.8 Hz, CH_2_N), 2.96 (2H, t, *J* = 7.2 Hz, CH_2_), 2.39 (3H, s, CH_3_); EIMS *m*/*z 404* [M]^+^ (1), *402* [M]^+^ (3), 195 (6), 193 (10), 191 (22), 180 (36), 178 (100), 149 (6), 143 (8), 142 (5), 115 (8). Anal. Calcd for C_18_H_15_ClN_4_O_5_: C 53.67; H 3.75; N 13.91%. Found: C 53.60; H 3.72; N 13.98%.

*N-[2-(7-Chloro-2-methyl-1H-indol-3-yl)ethyl]-2-methyl-1,3-benzoxazole-7-carboxamide (****68****)*

^1^H NMR (DMSO-*d*_6_) *δ* 10.85 (1H, s, NH), 7.82 (1H, s, NHCO), 7.75 (1H, d, *J* = 7.7 Hz, *H*_arom_), 7.70 (1H, d, *J* = 7.7 Hz, *H*_arom_), 7.44 (1H, d, *J* = 7.8 Hz, H-4), 7.36 (1H, t, *J* = 7.8 Hz,H-5'), 6.98 (1H, d, *J* = 7.6 Hz,H-6), 6.88 (1H, d, *J* = 7.6 Hz, H-5), 3.58 (2H, q, *J* = 6.7 Hz,CH_2_N), 2.98 (2H, t, *J* = 7.2 Hz, CH_2_), 2.59 (3H, s, CH_3_-2'), 2.41 (3H, s, CH_3_-2); EIMS *m*/*z 369* [M]^+^ (4), *367* [M]^+^ (12), 193 (27), 192 (13), 191 (99), 180 (26), 179 (13), 178 (100), 160 (33), 143 (13), 142 (9), 132 (24), 115 (11), 91 (8), 77 (7), 63 (12). Anal. Calcd for C_20_H_18_ClN_3_O_2_: C 65.31; H 4.93; N 11.42%. Found: C 65.38; H 4.95; N 11.32%.

*2,4-Dichloro-N-[2-(7-chloro-2-methyl-1H-indol-3-yl)ethyl]-5-[(dimethylamino)sulfonyl]benzamide (****69****)*

^1^H NMR (DMSO-*d*_6_) *δ* 10.65 (1H, s, NH), 8.39 (1H, s, NHCO), 7.91 (1H, s, H-6'), 7.64 (1H, s, H-3'), 7.41 (1H, d, *J* = 7.7 Hz,H-4), 6.96 (1H, d, *J* = 7.5 Hz,H-6), 6.90 (1H, t, *J* = 7.7 Hz, H-5), 3.47 (2H, q, *J* = 6.7 Hz, CH_2_N), 2.95 (2H, t, *J* = 7.2 Hz, CH_2_), 2.87 (6H, s, CH_3_), 2.44 (3H, s, CH_3_); EIMS *m*/*z 491* [M]^+^ (1), *489* [M]^+^ (3), *487* [M]^+^ (3), 193 (21), 192 (7), 191 (67), 180 (28), 179 (13), 178 (100), 143 (11), 115 (9). Anal. Calcd for C_20_H_20_Cl_3_N_3_O_3_S: C 49.14; H 4.12; N 8.60%. Found: C 49.00; H 4.10; N 8.53%.

*2,4-Dichloro-5-[(dimethylamino)sulfonyl]-N-{2-[2-methyl-7-(trifluoromethyl)-1H-indol-3-yl]ethyl}benzamide (****70****)*

^1^H NMR (DMSO-*d*_6_) *δ* 10.67 (1H, s, NH), 8.43 (1H, s, NHCO), 7.91 (1H, s, H-6'), 7.72 (1H, d, *J* = 7.9 Hz,H-4),7.64 (1H, s, H-3'), 7.25 (1H, d, *J* = 7.5 Hz,H-6), 7.05 (1H, t, *J* = 7.7 Hz, H-5), 3.48 (2H, q, *J* = 6.8 Hz, CH_2_N), 2.99 (2H, t, *J* = 7.2 Hz, CH_2_), 2.87 (6H, s, CH_3_), 2.46 (3H, s, CH_3_); EIMS *m*/*z 523* [M]^+^ (1), 521 [M]^+^ (1), 226 (26), 225 (100), 212 (90), 193 (11), 192 (78), 191 (19), 178 (11), 172 (16), 144 (8). Anal. Calcd for C_21_H_20_Cl_2_F_3_N_3_O_3_S: C 48.29; H 3.86; N 8.04%. Found: C 48.17; H 3.82; N 7.97%.

*2,4-Dichloro-5-[(cyclopropylamino)sulfonyl]-N-{2-[2-methyl-7-(trifluoromethyl)-1H-indol-3-yl]ethyl}benzamide (****71****)*

^1^H NMR (DMSO-*d*_6_) *δ* 11.07 (1H, s, NH), 8.77 (1H, t, *J* = 5.9 Hz,NHCO), 8.42 (1H, s, NHSO_2_), 7.96 (1H, s, H_arom_), 7.91 (1H, s, H_arom_), 7.77 (1H, d, *J* = 7.9 Hz,H-4), 7.32 (1H, d, *J* = 7.5 Hz,H-6), 7.11 (1H, t, *J* = 7.7 Hz, H-5), 3.43 (2H, q, *J* = 6.8 Hz, CH_2_N), 2.94 (2H, t, *J* = 7.2 Hz, CH_2_), 2.41 (3H, s, CH_3_), 2.22 (1H, s, CHN), 0.48 (2H, m, CH_2_), 0.40 (2H, m, CH_2_); EIMS *m*/*z*  226 (14), 225 (100), 213 (10), 212 (71), 193 (9), 192 (63), 172 (13), 144 (7), 91 (8). Anal. Calcd for C_22_H_20_Cl_2_F_3_N_3_O_3_S: C 49.45; H 3.77; N 7.86%. Found: C 49.38; H 3.74; N 7.82%.

*Methyl 4-{[2-(4-fluoro-2,7-dimethyl-1H-indol-3-yl)ethyl]carbamoyl}benzoate (****72****)*

^1^H NMR (DMSO-*d*_6_) *δ* 11.15 (1H, s, NH), 8.86 (1H, t, *J* = 5.9 Hz,NHCO), 8.04 (2H, d, *J* = 8.3 Hz, *H-*2',6'), 7.97 (2H, d, *J* = 8.3 Hz, *H-3*',5'), 6.66 (1H, dd, *J* = 11.0 Hz, *J* = 7.9 Hz,H-6), 6.61 (1H, dd, *J* = 8.0 Hz, *J* = 4.8 Hz,H-5), 3.89 (3H, s, OCH_3_), 3.39 (2H, m, CH_2_N), 3.00 (2H, t, *J* = 7.5 Hz, CH_2_), 2.62 (3H, s, CH_3_-7), 2.32 (3H, s, CH_3_-2); EIMS *m*/*z* 368 [M]^+^ (8), 189 (28), 177 (11), 176 (100), 174 (6), 163 (6). Anal. Calcd for C_21_H_21_ FN_2_O_3_: C 68.47; H 5.75; N 7.60%. Found: C 68.60; H 5.79; N 7.49%.

*N-[2-(4-Fluoro-2,7-dimethyl-1H-indol-3-yl)ethyl]-2-methylbenzamide (****73****)*

^1^H NMR (DMSO-*d*_6_) *δ* 11.15 (1H, s, NH), 8.37 (1H, t, *J* = 5.8 Hz, NHCO), 7.30 (2H, dd, *J* = 15.2 Hz, *J* = 7.5 Hz,H_arom_ ), 7.22 (2H, dd, *J* = 15.8 Hz, *J* = 7.5 Hz,H_arom_ ), 6.66 (1H, dd, *J* = 11.0 Hz, *J* = 8.0 Hz,H-6), 6.60 (1H, dd, *J* = 8.0 Hz, *J* = 4.8 Hz,H-5), 3.33 (2H, m, CH_2_N), 2.98 (2H, t, *J* = 7.5 Hz, CH_2_), 2.61 (3H, s, CH_3_-7), 2.35 (3H, s, CH_3_), 2.33 (3H, s, CH_3_); EIMS *m*/*z* 325 [M+1]^+^ (7), 324 [M]^+^ (32), 190 (8), 189 (54), 177 (12), 176 (100), 175 (5), 119 (15), 91 (15). Anal. Calcd for C_20_H_21_FN_2_O: C 74.05; H 6.52; N 8.64%. Found: C 74.16 H 6.56; N 8.54%.

*N-[2-(4-Fluoro-2,7-dimethyl-1H-indol-3-yl)ethyl]-3,4-dimethoxybenzamide (****74****)*

^1^H NMR (DMSO-*d*_6_) *δ* 11.13 (1H, s, NH), 8.51 (1H, t, *J* = 5.8 Hz,NHCO), 7.47 (1H, d, *J* = 8.4 Hz, *H-*6'), 7.44 (1H, s, H-2'), 7.02 (1H, d, *J* = 8.4 Hz, *H-*5'), 6.66 (1H, dd, *J* = 11.0 Hz, *J* = 8.0 Hz,H-6), 6.61 (1H, dd, *J* = 7.7 Hz, *J* = 4.9 Hz,H-5), 3.81 (3H, s, OCH_3_), 3.80 (3H, s, OCH_3_), 3.36 (2H, m, CH_2_N), 2.98 (2H, t, *J* = 7.5 Hz, CH_2_), 2.62 (3H, s, CH_3_-7), 2.32 (3H, s, CH_3_-2); EIMS *m*/*z* 371 [M+1]^+^ (7), 370 [M]^+^ (31), 190 (11), 189 (83), 177 (14), 176 (100), 175 (5), 165 (25), 79 (5). Anal. Calcd for C_21_H_23_ FN_2_O_3_: C 68.09; H 6.26; N 7.56%. Found: C 68.21; H 6.31; N 7.42%.
